# A systematic review of real-world gait-related digital mobility outcomes in Parkinson’s disease

**DOI:** 10.1038/s41746-025-01938-y

**Published:** 2025-09-30

**Authors:** Cameron Kirk, Emma Packer, Ashley Polhemus, Mhairi K. MacLean, Harry Bailey, Felix Kluge, Heiko Gaßner, Lynn Rochester, Silvia Del Din, Alison J. Yarnall

**Affiliations:** 1 Translational and Clinical Research Institute, Faculty of Medical Sciences, Newcastle University, Newcastle upon Tyne, UK; 2 Epidemiology, Biostatistics and Prevention Institute, University of Zurich, Zurich, Switzerland; 3 Department of Biomechanical Engineering, University of Twente, Twente, The Netherlands; 4 Novartis Institutes of Biomedical Research, Novartis Pharma AG, Basel, Switzerland; 5 Department of Molecular Neurology, University Hospital Erlangen, Erlangen, Germany; 6 Fraunhofer Institute for Integrated Circuits IIS, Erlangen, Germany; 7 National Institute for Health and Care Research (NIHR) Newcastle Biomedical Research Centre (BRC), Newcastle upon Tyne, UK; 8 Newcastle upon Tyne Hospitals NHS Foundations Trust, Newcastle upon Tyne, UK

**Keywords:** Medical research, Biomedical engineering

## Abstract

Clinical assessments of Parkinson’s disease (PD) focus on structured motor tasks, potentially underestimating real-world mobility impairment. Digital mobility outcomes (DMOs) from wearable devices, offer a promising alternative, but their clinical value remains unclear. This study reviewed the clinical utility of real-world DMOs in PD. Eight databases were searched (2000–2024). Eligibility included reports assessing real-world gait-based DMOs in PD. Study selection and data extraction were conducted independently by four reviewers. No meta-analysis was performed. Twenty-seven reports met inclusion criteria, reporting 21 DMOs. Walking speed was most common (92%), followed by step length (59%) and stride time (48%). Devices, assessment durations and walking bout definitions varied. DMOs differentiated PD from controls and real-world from supervised conditions. PD Subgroup analyses and associations with motor severity were inconsistent. Real-world DMOs offer valuable insight into PD mobility. Standardised protocols are needed for comparability. Further research should explore predictive utility and complex or composite DMOs. Trial Registration: PROSPERO 2025 CRD42021281213.

## Introduction

Parkinson’s disease (PD) is a progressive neurological disorder characterised by the cardinal motor symptoms of bradykinesia, rigidity and tremor^1,2^. The presence of these motor symptoms often manifest as mobility impairments which are detrimental to an individual’s health and quality of life^3^. Gait disturbances are among the most disruptive mobility impairments experienced by people with PD (PwP), which appear early, in the prodromal stages and deteriorate over time^4,5^. Thus, one of the key challenges for delivering effective management and treatment of PD is focused upon extension of quality of life through maintaining physical mobility.

Due to the heterogeneity in PD, accurately measuring and monitoring the influence of motor symptoms on mobility is challenging. Accurate measurement is further complicated by variability in symptom profiles between PwP and the tendency for motor symptoms to fluctuate throughout the day. In PwP, the Movement Disorder Society-Unified Parkinson’s Disease Rating Scale Part III (MDS-UPDRS III) remains the most widely used method to evaluate motor symptom severity^6^. However, the score is based on subjective visual interpretation, and the assessment is only accessible to those who are physically capable of travelling to a clinic, which increases the patient, clinician and carer burden. In a research setting, mobility is assessed across brief functional walking tasks (10 metre straight walk, timed up and go test, etc.) in supervised environments^7,8^. Additionally, these assessments capture ‘mobility capacity’, rather than ‘mobility performance’(e.g. walking performed in the real-world)^9^. Most fundamentally, existing supervised assessments such as the MDS-UPDRS III or scripted gait tasks do not directly assess the impact of symptoms upon everyday mobility in PD^8^. This limits understanding of how PD symptoms impact aspects of health which are of great importance to patients, such as preservation of their physical mobility in their everyday lives^10^.

With the development of wearable devices, walking speed and a range of clinically relevant gait characteristics, collectively referred to as digital mobility outcomes (DMOs)^11^, can now be quantitatively and continuously measured in real-world settings. DMOs enable remote monitoring of mobility, potentially improving access to assessment for individuals in isolated or underserved communities and thereby increasing inclusivity in clinical trials. DMOs offer a diverse profile of mobility assessed over thousands of walking bouts (WBs), which differ in their duration, context and purpose. WBs are defined as walking sequences containing at least two consecutive strides of both feet. Start and end of a WB are determined by a resting period or any other activity (non-walking period)^12^. These tools can complement existing methods of mobility assessment in PD and help address their limitations. A large scoping review^13^ demonstrated that DMOs assessed in supervised settings can distinguish PwP from controls and are associated with clinical measures of motor severity (e.g. MDS-UPDRS III). However, the clinical utility of DMOs measured in real-world environments has not been widely reviewed. It is therefore crucial to investigate how real-world DMOs can enhance PD clinical assessments. Polhemus et al.^13^ hypothesised that limited real-world research may stem from a lack of consensus on technically valid measurement protocols, hindering evidence comparability. Yet, since 2021, technological advances and increasing clinical interest may have led to a substantial rise in relevant publications.

The overarching aim of this review was to understand how real-world gait DMOs are currently measured and described in PwP. This review builds upon the scoping review of DMOs assessed in a supervised setting^13^, adopting a similar approach. We stratified this review into five core objectives described in Box 1.

Box 1 Objectives of this systematic review **Objective 1:** Provide an overview of the measurement approaches and protocols used to captures real-world DMOs in PwP

**Objective 2:** Understand whether DMOs are contextually sensitive in their difference between real-world and supervised assessments.

**Objective 3:** Establish discriminant ability of DMOs, through understanding whether real-world DMOs are commonly reported as different between PwP and controls.

**Objective 4:** Determine whether real-world DMOs can discriminate between specific PD sub-groups.

**Objective 5:** Explore construct validity of real-world DMOs through their reported associations with clinical measurements of motor severity.

## Results

**Fig. 1:**
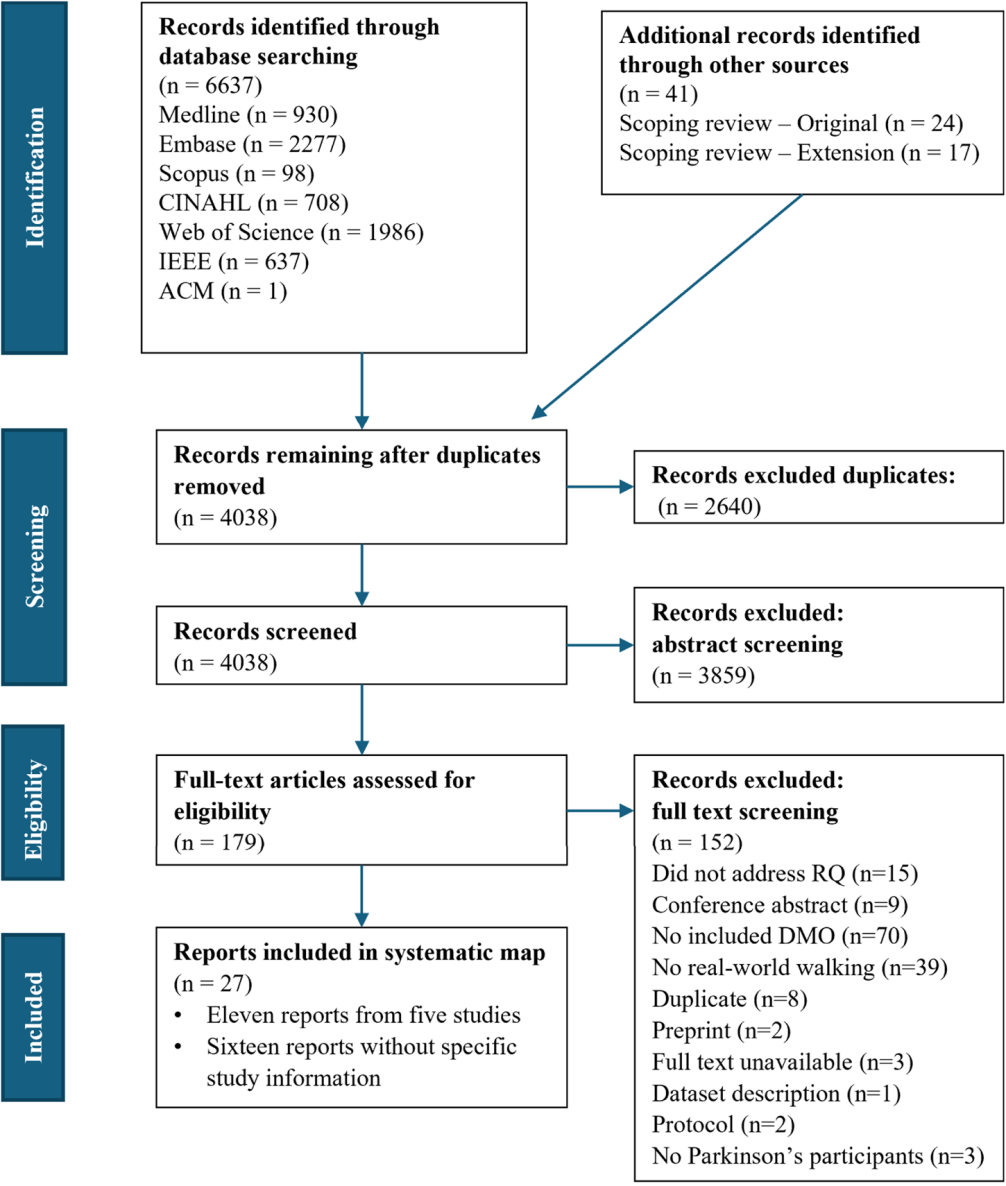
PRISMA flow diagram. This diagram outlines the screening and selection process for studies included in the review. The process adhered to the PRISMA 2020 guidelines. Abbreviations: RQ research question.

An initial search conducted in October 2021 identified 4411 reports. An updated search (April 2024) identified an additional 2226 reports, for a total of 6637 reports across the initial search and review update. The number of articles per exclusion reason can be found in Supplementary Fig. 1. A total of 27 reports were included in this review, with 11 reports including participants from five studies and 16 reports not detailing which study participants were recruited from (Fig. 1). A list of the reports excluded in the full-text review can be found in Supplementary Table 1.

Overall, the quality of reports was moderate [median = 12, Range = 8:15], quality scores reported across each individual report can be found in Supplementary Tables 3–4.

### Objective 1: Characteristics and measurement approach of included reports

Reports included a median of 47 PwP, with a range of 11–587 individuals. Age ranged between 60 and 72 years and MDS-UPDRS III between 12.8 and 46.2 points.

Heterogeneity was demonstrated in the measurement protocols for capturing DMOs, with various differences in the location, attachment, number of devices, length of assessment and WB thresholds. (Table 1). Specifically, across the included reports eight different wearable device combinations were applied to monitor DMOs. A single device worn on the lower-back was the most commonly adopted (*n* = 10), followed by devices worn on the lower back and feet (*n* = 7) and an individual foot (*n* = 4) (Table 1). Wearable devices were most commonly attached to the body with adhesives and bandages (*n* = 5), with 10 reports not reporting sensor attachment methods. Only one report utilised multiple wearable devices, namely a smart phone and a wrist-worn device^14^. The most common length of assessment was seven-days (*n* = 15), and the most commonly used WB threshold was any WB over 60 s (Table 1).

**Table 1:** Measurement protocols and data aggregation methods applied in real-world studies

Instrument	Number (%)
Smart phone^14^	1 (4%)
Wearable device (foot)^15,16,28,31^	4 (14%)
Wearable device (lower back)^17,18,21,27,30,34–38^	10 (37%)
Wearable device (lower back & feet)^19,22,23,29,32,33,39^	7 (25%)
Wearable device (shirt pocket)^20^	1 (4%)
Wearable device (thigh, forearm and trunk)^26^	1 (4%)
Wearable device (wrist)^14^	1 (4%)
Wearable device (waist)^24,25^	2 (7%)
**Sensor attachment**	**Number (%)**
Adhesive and bandage^17,21,27,37,38^	5 (18%)
Instrumented socks and elastic belt^19,29,33,39^	4 (14%)
Shoe clip^28,31^	2 (7%)
Unreported^15,16,18,22–26,32,35^	10 (37%)
Velcro belt^30,34,36^	4 (14%)
Wrist strap^14^	1 (4%)
Worn in pocket^20^	1 (4%)
**Length of assessment (days)**	**Number (%)**
1^15,20,24,25^	4 (14%)
3^30,34,36^	3 (11%)
4^35^	1 (4%)
7^14,17–19,21,22,27,29,32,33,37–39,44^	15 (55%)
12^16^	1 (4%)
14^28,31^	2 (7%)
**Walking bout duration**	**Number (%)**
Unreported^14,24–26,29–31,34,39^	9 (33%)
WBs < 10 s^17,18^	2 (7%)
10–20 s^17^	1 (4%)
15–30 s^16^	1 (4%)
20–30 s^17^	1 (4%)
10–30 s^18,21^	2 (7%)
30–60 s^16–18,21^	4 (14%)
WBs > 30 s^38^	1 (4%)
60–120 s^17,18^	2 (7%)
WBs > 60 s^16,18,21,27,34,37^	6 (22%)
WBs > 120 s^17,18,27^	3 (11%)
All WBs > 3 s^19,20,23,32^	4 (14%)
All WBs > 10 s^21,27,33^	3 (11%)
All WBs > 15 s^15^	1 (4%)
>5 strides^28^	1 (4%)
Between 4 and 19 steps^35^	1 (4%)
>20 steps^35^	1 (4%)

*WB* walking bout.

**Fig. 2:**
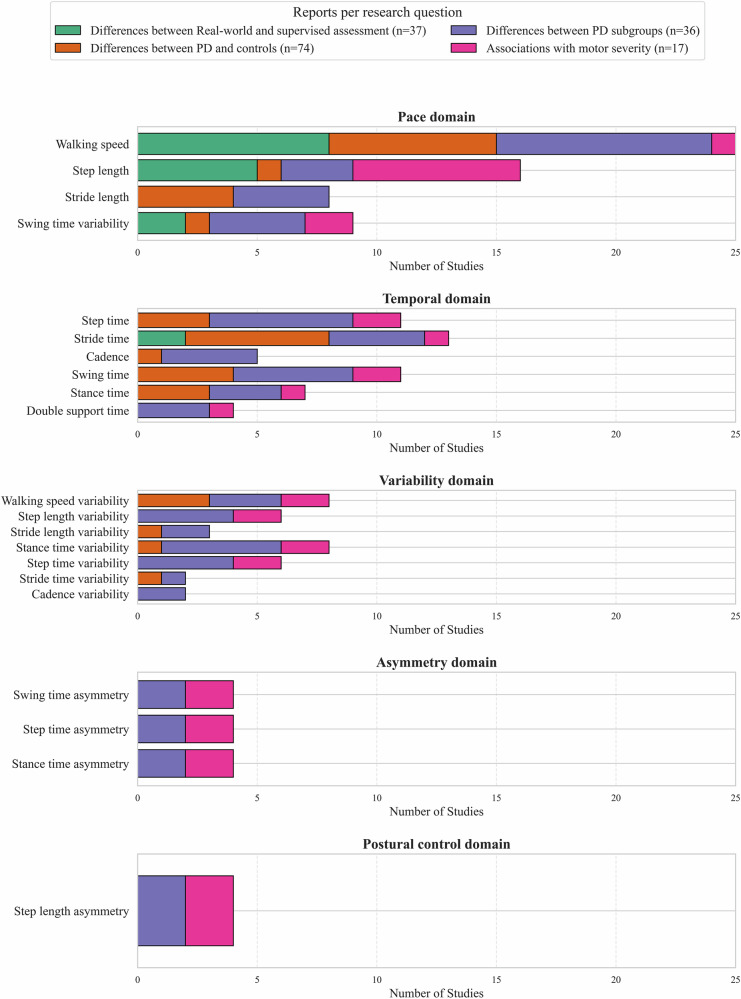
Distribution of digital mobility outcome (DMO) reports across five gait domains^77,79^, grouped by research question. This figure illustrates the number of study reports evaluating digital mobility outcomes (DMOs) within five key gait domains: Pace, Temporal, Variability, Asymmetry and Postural Control. Horizontal bar charts represent the number of reports for each DMO variable (e.g. walking speed, stride length, swing time variability), stratified by the primary research question addressed. These questions include: (1) differences between real-world and supervised assessments (green), (2) differences between people with Parkinson’s disease (PD) and control groups (orange), (3) differences between PD subgroups (blue), and (4) associations between DMOs and motor severity (pink).

Differences between PwP and healthy control participants was the most widely explored objective (11 reports), DMOs of the pace domain were explored in the largest majority of reports. The total number of reports across each DMO and research question is displayed in Fig. 2.

### Objective 2: DMOs assessed in real-world compared to supervised settings

Six reports explored whether DMOs were different between real-world and supervised assessment^15–20^. The quality scores of the included studies ranged from 8 to 13, with a median of 12, indicating strong overall methodological rigour. Common strengths across all reports included clearly defined populations and consistent implementation of validated outcome measures. However, information bias was a notable concern: only two reports (16%) detailing only >3 days were included^17,19^, and specified sensor attachment methods^17,20^.

The median sample size was 27, and PwP had a pooled mean age of 69.1 years and pooled mean MDS-UPDRS III was 30.9 points. One report had a median age and MDS-UPDRS III of 69 years and 22 points respectively.

All reports utilised the same devices for both real-world and supervised assessments. Two reports assessed a similar sample of participants recruited from the same population (ICICLE-GAIT) with a single lower-back worn device^17,18^; two reports utilised a single foot-worn device^15,16^; one report had three devices worn on the feet and lower back^19^; and one report had five devices worn on the thighs, shanks and trunk^20^. An overview of the identified supervised assessment methods can be found in Supplementary Table 6.

In the real-world setting, three reports recorded for 7-days^17–19^, two reports assessed participants across a single day^15,20^ and one report recorded for 12 days^16^. For the supervised protocols, all reports included straight walking assessment within their approach. Distances ranged from 7 to 20 m, with four reports also including curvilinear walking or turning as part of the task^15,16,18,19^ (Table 2).

**Table 2:** Details and outcome of each included study in this review that compared DMOs between real-world and supervised assessment

Study	Population characteristics (Number, age, motor severity)	Supervised measurement protocol	Real-world measurement protocol	DMO	Statistics	Outcome
Atrsaei et al.^15^	27 PD, Age: 70 ± 7.7 years, MDS-UPDRS III: 25 ± 11.8 points.	Single device worn on foot (RehaGait, Hasomed GmbH, DE). Protocol: 20-m straight walk test at convenient and fast speed + circular walking tests (1080° around a circle) at left and right directions.	Single device worn on foot (Physilog®5, Gait Up, CH), worn for one day. DMOs aggregated across all walking bouts > 3 s.	Walking speed extracted from each peak of a bimodal distribution.	Two-sided Wilcoxon rank sum test, correlation.	Lowest and highest bimodal peak of real-world walking speed not significantly different (*p* > 0.05) to corresponding peak in supervised setting.
Corrà et al.^16^	27 PD, Age: 69 [64–73] years, MDS-UPDRS III: 22 [15, 31] points.	Single device worn on foot (RehaGait, Hasomed GmbH, DE). Protocol: Straight walk (20-m, 2 trials)/Circular walk (1.2 m diameter, 3 trials both directions). Repeated on and off medication.	Single device worn on foot (Physilog®5, Gait Up, CH). Worn for 12 days. DMOs Aggregated across WB durations: >15 s, 15–60 s, 30–60 s, >60 s.	Maximum, 90th, 75th, 50th and 25th percentile and median of walking speed measured separately for ON and OFF medication states in both real-world and supervised assessment.	Pearson or Spearman for parametric or non-parametric data.	Faster real-world walking speeds (higher percentiles) correlated with supervised walking speed in ON state (*p* < 0.001, *r* = 0.64). Slower real-world walking speeds (lower percentiles) correlated with supervised walking speed in OFF medication (*p* = 0.030, *r* = 0.15).
Del Din et al.^17^	50 PD, Age: 69.1–(8.3) years, MDS-UPDRS III: 32–(10) points.	Single device worn on the lower back (Axivity AX3, UK). Protocol: 10 m straight walks, 4 trials.	Single device worn on the lower back (Axivity AX3, UK). Attached with adhesive patch and tape. Worn for 7 days. DMOs WB aggregated across durations: <10, 10–20, 20–30, 30–60, 60–120 and >120 s.	Walking speed (mean, variability), step length, step time, swing time and stance time (mean, variability, asym’metry).	Mann-Whitney *U* test.	Decreased pace, increased rhythm, higher variability and asymmetry in real-world in comparison to supervised setting (*P* ≤ 0.001). Differences were greater as WB duration increased.
Rehman et al.^18^	99 PD, Age: 68.3 ± 8.9 years, MDS-UPDRS III: 31.53 ± 9.79 points.	Single device worn on lower back (Axivity AX3, UK). Protocol: Continuous walk around an oval circuit.	Single device worn on lower back (Axivity AX3, UK). Worn for 7 days. DMOs aggregated across WB durations: <10, 10 ≤ WBs ≤ 30 s, 30 ≤ WBs ≤ 60 s, 60 ≤ WBs ≤ 120 s, WBs > 60 s, WBs > 120 s.	Walking speed (mean, variability), step length, step time, swing time and stance time (mean, variability, asymmetry).	Mixed ANOVA.	Shorter step time and longer step length compared to real-world across each duration threshold. Gait variability (swing time, step time, stance time, step length and step velocity) reduced when measured in supervised setting. Gait was more symmetrical in supervised setting.
Shah et al. ^19^	16 PD, Age: 70.5 ± 5.1 years, MDS-UPDRS III: 30.44 ± 10.76 points.	Three devices; two instrumented socks and one wearable on lower back (Opals by APDM Wearable Technologies, USA). Protocol: 7-metre walk with 180-degree turn.	Three devices; two instrumented socks and one wearable on lower back (Opals by APDM Wearable Technologies, USA). Worn for 7 days. DMOs aggregated across all walking bouts.	Cadence (Steps/mins), double support (%), Walking speed (m/s), stride duration (s), stride length (m) and swing (%).	Paired Wilcoxon testing.	Walking speed and swing duration significantly lower in daily life (*p* < 0.001) and double support significantly longer.
Toosizadeh et al.^20^	15 PD, Age: 71.2 ± 6.3 years, MDS-UPDRS III: 34.8 ± 10.9 points.	Five devices worn on shanks, thighs and trunk (LEGSys and BalanSens, BioSensics, USA). Protocol: Straight walk.	Single wearable device worn in shirt pocket (PAMSys, BioSensics, USA). Worn for one day. DMOs aggregated across all walking bouts > 3 s.	Walking speed and stride length.	Correlations between in-clinic and in-home parameters assessed by linear regression-analysis of variance (ANOVA) model for walking speed and reported as Pearson correlations (R).	Walking speed was different between real-world and supervised assessment, evidenced by no significant correlations.

Data presented as mean-(SD), or median-[IQR].

In the majority of reports (*n* = 5), DMOs were reported as significantly different between real-world and supervised assessments. Reports typically reported lower values in real-world settings and most commonly reporting slower walking speeds and reduced step or stride lengths compared to supervised assessments (Table 2, column 7). Two reports compared extreme values, finding significant correlations between the ‘fastest’, or ‘highest’ regions of the distribution of walking speed between real-world and supervised assessments^15,16^.

### Objective 3: Differences between PD and controls

Eleven studies investigated whether DMOs differed between PwP and healthy controls^14,17–26^. Quality scores of objective three ranged from 8 to 13, with a median of 12, indicating excellent quality. Selection bias was most strongly addressed, as majority of reports clearly defined populations with validated measures consistently implemented. Information bias was inconsistent with only five studies (50%) describing device attachment^17,21,22,24,25^ and whether >3 days of data capture was controlled for^14,17,19,21,22^.

Across these studies, the median sample size was 27 for control participants and 47 for PwP. The pooled mean age was similar between groups: 67.84 years for controls and 67.62 years for PwP. MDS-UPDRS Part III had a pooled mean score of 26.5 points among PwP.

Devices were placed across six locations, specifically: three reports assessed participants using a single lower-back-worn device recruited from the same population (ICICLE-GAIT)^17,18,21^; three reports employed three devices worn on the feet and lower back^19,22,23^; two reports used a single anterior waist-worn device^24,25^; one report used a single shirt-pocket-worn device^20^; one report used five devices placed on the thighs, forearms and trunk^26^; and one report placed a smartphone on the lower back^14^. Seven reports assessed participants over seven days^14,17–19,21–23^, while four reports had shorter durations with two reports recording over 24 h^20,24^, one report over 25 h^25^ and one report over 45 h^26^ (Table 3).

**Table 3:** Differences between Parkinson’s and controls: Details and outcome of each included study in this review that compared DMOs between people with PD and control participants

Study	Healthy control characteristics (Number, Age)	PD Characteristics (Number, Age, Motor severity)	Real-world measurement approach	DMOs	Statistical comparison	Outcome
Adams et al.^26^	17 participants, Age: 64 (9) years.	17 participants, Age: 66 (11) years, MDS-UPDRS III: 14 (10) points.	Five devices worn on each thigh and forearm, and one on the trunk (BioStampRC®, USA). Worn for 45 h. DMOs aggregated across all WBs.	Step length, step duration and walking speed.	Wilcoxon rank-sum test.	No significant difference in step length, walking speed, or step duration.
Adams et al.^14^	50 participants, Age: 60 (9.2) years.	82 participants, Age: 63 (9.4) years, MDS-UPDRS III: 24.1 (10.2) points.	Single smartphone wearable device worn on the lower back via a pouch (iPhone 10 or 11, Apple Inc., USA). Worn for 7 days. DMOs aggregated from all WBs.	Stance duration, cadence, double support duration and walking speed.	Wilcoxon rank-sum test.	People with PD had increased stance time (*p* = 0.007), slower cadence (*p* = 0.005) and increased double support time (*p* = 0.006). Walking speed did not significantly differ between groups (*p* = 0.13).
Del Din et al.^17^	50 participants, Age: 69.8 (7.2) years.	50 participants, Age: 69.1 (8.3) years, MDS-UPDRS III: 32 (10) points.	Single device worn on the lower back (Axivity AX3, UK). Attached with adhesive patch and tape. Worn for 7 days. DMOs aggregated across WB durations: <10, 10–20, 20–30, 30–60, 60–120 and >120 s.	Walking speed (mean, variability), step length, step time, swing time and stance time (mean, variability, asymmetry).	*T*-tests or Mann-Whitney *U* tests.	Step velocity and step length significantly lower in PD (*p* ≤ 0.001). Significantly larger step length asymmetry (*p* = 0.004) and swing time (*p* = 0.008) in PD. Shorter WBs <10 s showed no difference. WBs 30–60 s showed slower pace and increased asymmetry in PD. Variability differences evident in WBs >60 s.
Kirk et al.^21^	94 participants, Age: 72 (6) years.	62 participants, Age: 69 (10) years, MDS-UPDRS III: 38 (12) points.	Single device worn on the lower back (Axivity AX3, UK). Attached with adhesive patch and tape. Worn for 7 days. DMOs aggregated across WB durations: >10 s, 10–30 s, 30–60 s and > 60 s.	Walking speed.	Wilcoxon-H test or *t*-test.	Significantly slower walking speed in PD across each WB duration threshold (*p* < 0.05).
Rehman et al.^18^	52 participants, Age: 68 (9) years.	99 participants, Age: 68.3 (8.9) years, MDS-UPDRS III: 31.53 (9.79) points.	Single device worn on the lower back (Axivity AX3, UK). Attached with adhesive patch and tape. Worn for 7 days. DMOs aggregated across WB durations: <10 s, 10–30 s, 30–60 s, 60–120 s, >120 s.	Walking speed (mean, variability), step length, step time, swing time and stance time (mean, variability, asymmetry).	Mixed ANOVA.	People with PD walked slower, with shorter step length and slower step time. Lower swing time variability (*p* = 0.033), higher swing time asymmetry (*p* = 0.006). Walking speed and step length differences significant for WBs >10 s. Asymmetry and variability differences depended on WB length.
Shah et al.^22^	27 participants, Age: 64.4 (7.5) years.	29 participants, Age: 67.7 (5.3) years, MDS-UPDRS III: 34.6 (11) points.	Three devices: two instrumented socks and one wearable on lower back (Opals by APDM Wearable Technologies, USA). Worn for 7 days. DMOs aggregated across all walking bouts.	Walking speed, stride length, cadence, step duration, swing time (mean, variability).	Mann-Whitney *U* test.	Walking speed, stride length and swing time significantly lower in PD (*p* < 0.05). Variability of all DMOs significantly larger in PD (*p* < 0.05).
Shah et al.^23^	20 participants, Age: 66.8 (7.16) years.	29 participants, Age: 66.8 (7.1) years, MDS-UPDRS III: not reported.	Three devices: two instrumented socks and one wearable on lower back (Opals by APDM Wearable Technologies, USA). Worn for 7 days. DMOs aggregated across all WBs.	Walking speed, cadence, stride length, swing %, double support % (mean, variability).	ROC curve (threshold >0.80 for discriminative effect).	The only DMOs with discriminative effect between groups was stride length variability (AUC = 0.81) and swing time variability (AUC = 0.84).
Shah et al.^19^	15 participants, Age: 67 (7) years.	16 participants, Age: 75 (5) years, MDS-UPDRS III: 30.4 (10.7) points.	Three devices: two instrumented socks and one wearable on lower back (Opals by APDM Wearable Technologies, USA). Worn for 7 days. DMOs aggregated across all WBs.	Cadence, double support, Walking speed, stride duration, stride length, swing %.	Paired Wilcoxon testing.	Significantly lower walking speed (*p* = 0.003) and stride length (*p* = 0.006) in PD. No difference in cadence, stride duration, swing time, or double support.
Terashi et al.^25^	17 participants, Age: 64.6 (4.4) years.	64 participants, Age: 67.5 (5.7) years, MDS-UPDRS III: 19.0 (7.4) points.	Single device worn on the anterior waist (Portable Gait Rhythmogram—PGR). Worn for 25 h. DMO aggregated across all WBs.	Stride time.	Student’s *t*-test.	No difference between groups.
Terashi et al.^24^	15 participants, Age: 67.9 (4.7) years.	106 participants, Age: 68 (6.7) years, MDS-UPDRS III: 17.3 (9.0) points.	Single wearable device worn on anterior waist (MIMAMORI-Gait, LSI Medience Corporation, Japan). Worn for 24 h. DMOs aggregated across all WBs.	Stride time.	Student’s *t*-test.	No difference between groups.
Toosizadeh et al.^20^	35 participants, Age: 71.9 (3.8) years.	15 participants, Age: 71.2 (6.3) years, MDS-UPDRS III: 34.8 (10.9) points.	Single wearable device worn in the shirt pocket (PAMSys, BioSensics, USA). Worn for one day. DMOs aggregated across all walking bouts >3 s.	Walking speed.	Unpaired *t*-test, Mann-Whitney *U* test.	No difference between groups.

Data presented as mean-(SD), or median-[IQR].

DMOs from the pace domain were the most frequently reported, appearing in nine reports, with five reporting significant differences between PD and control groups. PwP typically walked with slower walking speeds and step or stride lengths in comparison to controls. Nine reports examined DMOs from the rhythm domain, with five finding significant differences, with PwP typically having longer step or stride times. However, three reports observed no significant difference in stride time^19,24,25^. Four reports analysed variability, all reporting significantly higher variability in PD^17,18,22,23^. Two reports investigated asymmetry, with significant differences observed depending on the walking bout threshold^17,18^ (Table 3).

### Objective 4: Differences between PD sub-groups

Nine reports compared DMOs between PD sub-groups, specifically: five reports compared between faller status^27–31^; three compared between individuals with and without freezing of gait (FoG) symptoms^32–34^, and one report assessed differences between individuals with and without fatigue symptoms^35^. The included reports had a median quality score of 12, ranging from 8 to 15, indicating excellent overall quality. As with previous objectives, populations were clearly defined, main outcomes were well described, and validated measures were consistently implemented across the majority of reports. However, confounding bias was a notable limitation: 5 reports (55%)^28,30,33,34,36^ did not adjust for confounding variables in their statistical analyses, potentially affecting the internal validity of the observed associations.

Among studies investigating falls, people with Parkinson’s who had experienced falls had a higher pooled mean age (69.45 years) compared to non-fallers (64.51 years). Motor severity, measured by the MDS-UPDRS Part III, was similar between groups, with pooled means of 29.68 points for fallers and 29.03 points for non-fallers. In studies examining FoG, individuals with FoG symptoms had a comparable age (pooled mean = 67.41 years) and motor severity (MDS-UPDRS III pooled mean = 40.16 points) to those without FoG symptoms (67.76 years; 40.2 points, respectively). In the fatigue study, participants with fatigue were older (69.6 years) than those without fatigue (64.9 years) and had slightly higher motor symptom severity (15.9 vs. 12.8 MDS-UPDRS III points).

In terms of measurement approaches, four reports used a single lower-back-worn device^27,30,34,35^ and three reports used three devices placed on the feet and lower back^29,32,33^., and two reports used a two feet-worn devices^28,31^. Four reports collected data over 7 days^27,29,32,33^, two reports over 14 days^28,31^, two reports over 3 days^30,34^ and one report over 4 days^35^ (Table 4).

**Table 4:** Differences between PD subgroups: Details and outcome of each included study in this review that compared DMOs between people with PD and control participants

Study	Group comparison	PD Group 1 characteristics (Number, Age, Motor severity)	PD Group 2 characteristics (Number, Age, Motor severity)	Real-world measurement approach	DMOs	Statistical method	Outcome
Del Din et al.^27^	Fallers vs non-fallers (fallers: at least 2 falls in 6 months prior to assessment).	155 PD Fallers, Age: 71.5 (6.4) years, MDS-UPDRS III: 28.6 (5.7) points.	15 PD Non-fallers, Age: 64.5 (8.6) years, MDS-UPDRS III: 31.4 (13.2) points.	Single device worn on lower back (Axivity AX3, UK), worn for 7 days. DMOs aggregated across WB durations: across WBs: >10 s, >60 s and >120 s.	Walking speed (mean, variability), step length, step time, swing time and stance time (mean, variability, asymmetry).	General linear modelling.	Step velocity, step length and step length variability significantly different (*p* < 0.05) between fallers and non-fallers.
Roth et al.^28^	Fallers vs non-fallers (fallers: at least 1 fall in 3-month follow-up after 2-week recording).	11 PD Fallers, Age: 64.1 (8.3) years, UPDRS III: 13.4 (6.2) points.	27 PD Non-fallers, Age: 65.2 (8.1) years, MDS-UPDRS III: 21.3 (8.8) points.	Two devices worn on feet via shoe clips (Mobile-GaitLab, Portabiles Healthcare Technologies, DE), worn for 14 days. DMOs aggregated across all WBs.	Stride, stance, swing times, stride length, walking speed (mean and variability).	Non-parametric Mann-Whitney *U.*	No significant difference in any DMO between groups.
Shah et al.^29^	Fallers vs non-fallers (fallers: at least 1 fall in the prior 6 months).	17 PD Fallers, Age: 68.7 (11.1) years, MDS-UPDRS III: 46.1 (10) points.	17 PD Non-fallers, Age: 66.82 (6.6) years, MDS-UPDRS III: 43.8 (11.3) points.	Three devices: two instrumented socks and one wearable on lower back (Opals by APDM Wearable Technologies, USA), worn for 7 days DMOs aggregated across all WBs.	Walking speed, walking speed variability, stride length/time, double support %, stride time/length variability, swing time, cadence.	Mann–Whitney *U* test.	No significant differences between fallers and non-fallers.
Ullrich et al.^31^	Fallers vs non-fallers (fallers: self-reported fall events in a 3-month follow-up).	10 PD Fallers, Age: 64.5 (8.2) years, MDS-UPDRS III: 21.7 (9.1) points.	25 PD Non-fallers, Age: 63.6 (8.4) years, MDS-UPDRS III: 12.9 (6.2) points.	Two devices worn on feet via shoe clips (Mobile-GaitLab, Portabiles Healthcare Technologies, DE), worn for 14 days. DMOs aggregated across all WBs.	Walking speed, stride length, stride time, swing time, stance time.	Non-parametric Mann-Whitney *U.*	All gait parameters significantly lower in prospective fallers (*p* < 0.001).
Weiss et al.^30^	Fallers vs non-fallers (fallers: assessed via fall calendars, 1-year follow-up).	40 PD Fallers, Age: 66.5 (8.2) years, MDS-UPDRS III: 33.4 (11.4) points.	67 PD Non-fallers, Age: 64 (9.8) years, MDS-UPDRS III: 33.9 (12.6) points.	Single device worn on lower back (Dynaport, McRoberts, NE), worn for 3 days DMOs aggregated across all WBs.	Cadence.	Paired testing.	No significant difference between groups.
Mancini et al.^33^	FOG vs non-freezing of gait symptoms (FOG-Q classification).	14 PD with FOG MDS-UPDRS III: 36.1 (4) points.	10 PD without FOG, MDS-UPDRS III: 35.6 (3.9) points.	Three devices: two instrumented socks and one wearable on lower back (Opals by APDM Wearable Technologies, USA), worn for 7 days. DMOs aggregated across WBs >10 s.	Walking speed.	Independent *T*-tests.	No significant difference between cohorts.
Mancini et al.^32^	FOG vs non-freezing of gait symptoms (FOG-Q classification).	40 PD with FOG, Age: 69.6 (7.2) years, MDS-UPDRS III: 40.4 (8) points.	43 PD without FOG, Age: 69.05 (5.9) years, MDS-UPDRS III: 39.5 (10.8) points.	Three devices: two instrumented socks and one wearable on lower back (Opals by APDM Wearable Technologies, USA), worn for 7 days. DMOs aggregated from all WBs >3 s.	Walking speed.	Linear mixed model.	No significant difference between cohorts.
Weiss et al.^34^	FOG vs non-freezing of gait symptoms (FOG-Q classification).	28 PD with FOG, Age: 64.3 (8.7) years, MDS-UPDRS III: 46.2 (12.2) points.	44 PD without FOG, Age: 66.5 (8.7) years, MDS-UPDRS III: 42.2 (12.8) points.	Single device worn on lower back (Dynaport, McRoberts, NE), worn for 3 days DMOs aggregated across all WBs.	Cadence.	Paired testing.	No significant difference between cohorts.
Pilotto et al.^35^	People with and without fatigue symptoms (assessed using the PD Fatigue Scale, PFS-16).	21 PD with fatigue, Age: 69.6 (8) years, MDS-UPDRS III: 15.9 (9.5) points.	21 PD without fatigue, Age: 64.9 (8) years, MDS-UPDRS III: 12.8 (5.6) points.	Single device worn on lower back (Movisens GmbH, DE), worn for 4 days DMOs aggregated across steps: Shor (4–19 steps) and long (>20 steps).	Step time, step time variability, step time asymmetry (short WBs: 4-19 steps; long WBs: >20 steps).	Mann-Whitney nonparametric analysis (adjusted for age, sex, height, disease duration).	Significant differences in step time (*p* = 0.007), step time variability (*p* = 0.004) and step time asymmetry (*p* = 0.004) lower in non-fatigue group. Differences more pronounced at longer bout durations.

Data presented as mean-(SD), or median-[IQR].

Two reports found no differences in DMOs between fallers^28,29^. One report found that step velocity and step length, and step length variability were significantly lower and higher in fallers respectively^27^. Another report observed that walking, stride length, stride time, swing time and stance time were significantly lower in fallers^31^. There was no significant difference in walking speed or cadence between freezers and non-freezers. In comparison to individuals without fatigue symptoms, fatigued PD had significantly higher step time, step time variability and step time asymmetry, with differences more pronounced across longer bouts^35^ (Table 4).

### Objective 5: Associations with motor severity

Nine reports explored associations between DMOs and clinical scales of motor severity and motor disability. Six reports examined associations with MDS-UPDRS III score^20,21,24,37–39^; two reports explored associations with the PIGD subscore^23,39^, one report explored associations with the MDS-UPDRS III gait item score^16^ and one report across Hoehn and Yahr stages^25^.

Quality of objective five can be considered excellent with an average score of 12 and range between 8: 15. Most studies clearly defined populations, stated objectives and implemented validated measures consistently. However, risk of information and analytical bias was evident: only 2 studies controlled for >3 days (22%)^21,38^.

PwP had a pooled mean age and MDS-UPDRS III of 68.44 years, and 26.24 respectively. One report utilised median values reported median age of 69 years and median MDS-UPDRS III of 22 points. Three reports assessed participants using a single lower-back-worn device^21,37,38^, two reports used three devices (feet and lower back)^23,39^, one report used a single shirt pocket-worn device^20^, two reports used a single anterior waist-worn device^24,25^ and one report wore a single device on the foot^16^. Five reports assessed for 7 days^21,23,37–39^, two reports for 24 h^20,24^, one report for 12 days^16^ and one report for 25 h^25^.

The only DMOs associated with MDS-UPDRS III were walking speed across three reports^21,38,39^ and step length within one report^38^. Only the 90th and 25th percentile of walking speed was associated with the MDS-UPDRS III gait item when measured on and off medication, respectively^16^. One report found that step length and swing time variability were associated with the PIGD subscore^23^. Another report found PIGD score was significantly correlated with walking speed^39^.

One report explored differences between Hoehn and Yahr stages^25^ finding that walking speed and stride time were significantly faster for individuals with lower motor disability (Stage 1.5) in comparison to stages 2.5 and 3.5 (Table 5).

**Table 5:** Associations with motor severity: Details and outcome of each included study in this review that explored associations between DMOs with clinical measures of motor severity

Study	Clinical Measure	PD Characteristics	Real-world protocol	DMOs	Statistical measure	Outcome
Corrà et al.^16^	MDS-UPDRS III-gait item (item-3.1).	27 PD, Age: 69-[64-73] years, MDS-UPDRS III 22-[15, 31] points.	Single device worn on foot (Physilog®5, Gait Up, CH). Worn for 12 days. DMOs Aggregated across WBs >15 s, between 15–60 and 30–60 s and >60 s.	Maximum, 90th, 75th, 50th and 25th percentile and median of walking speed measured separately for ON and OFF medication states.	Pearson correlation for parametric data, Spearman correlation for non-parametric data.	Walking speed— OFF: No significant correlations, except Item 30 with 25th home-percentile, *R* = −0.44, *P* = 0.028. Walking speed - ON: No significant correlations, except Item 30 with 25th home-percentile, *R* = −0.61, *P* = 0.001.
Galperin et al.^37^	MDS-UPDRS III.	125 PD, Age: 71.5-(6.4) years, MDS-UPDRS III: 30.4, 13 points.	Single device worn on the lower back (Axivity AX3, UK). Attached with adhesive patch and tape. Worn for 7 days. DMOs estimated from WBs >60 s.	Step length (Mean).	Linear regression.	Step length: No significant association with MDS-UPDRS III.
Kirk et al.^21^	MDS-UPDRS III.	62 participants, Age: 69-(10) years, MDS-UPDRS III: 38-(12) points.	Single device worn on the lower back (Axivity AX3, UK). Worn for 7 days. DMOs estimated from WBs durations: > 10 s, 10–30 s, 30–60 s and >60 s.	Walking speed.	Linear regression.	Walking speed only associated with MDS-UPDRS III when aggregated from walking bouts >30–60 s (*p* = 0.047).
Mirelman et al.^38^	MDS-UPDRS III.	587 participants, Age: 67.9-(8.5) years, MDS-UPDRS III: 26-(13) points.	Single device worn on the lower back (Axivity AX3 or AX6, UK). Worn for 7 days. DMOs estimated from all WBs >30 s.	Step length, walking speed, stride time, step time, step time variability, step length variability, cadence.	Machine learning models. Five fold cross-validation matrix. Pearson’s R-squared as the performance metric.	Strongest predictors of MDS-UPDRS III score were step length (selection Score=10.11) and 90th percentile walking speed (selection score = 5.62).
Safarpour et al.^39^	MDS-UPDRS III & PIGD score.	31 participants, Age: 68.9–(5.9) years, MDS-UPDRS III: 32-(10) points.	Three devices; two instrumented socks and one wearable on lower back (Opals by APDM Wearable Technologies, USA). Worn for 7 days. DMOs estimated from all WBs.	Walking speed.	Spearman correlation coefficient.	Walking speed significantly correlated with both MDS-UPDRS III and PIGD sub-score (*p* < 0.05).
Shah et al.^23^	MDS-UPDRS III & MDS-UPDRS III PIGD sub-score.	29 participants, Age: 66.8–(7,1) years, MDS-UPDRS III: not reported.	Three devices; two instrumented socks and one wearable on lower back (Opals by APDM Wearable Technologies, USA). Worn for 7 days. DMOs estimated from all WBs.	Swing % [variability], Stride length [mean].	Spearman’s correlation.	PIGD score significantly correlated with Swing variability (*p* < 0.011, *R* = 0.46) and Stride length (*p* = 0.018). No associations with total MDS-UPDRS III score.
Terashi et al. ^25^	Participants across Hoehn and Yahr stages (modified H&Y stage).	64 participants, Age: 67.5 (5.7) years, MDS-UPDRS III: 19.0 (7.4) points.	Single device worn on anterior waist (PGR, portable gait rhythmogram), worn for 25 h DMO aggregated across all WBs.	Walking speed, stride time.	Student’s *t*-test.	Mean stride time was significantly faster in H&Y stage 1.5 in comparison to stages 2.5–3 (*p* ≤ 0.05).
Terashi et al. ^24^	MDS-UPDRS III.	106 participants, Age: 68-(6.7) years, MDS-UPDRS III: 17.3-(9.0) points.	Single wearable device worn on anterior waist (MIMAMORI-Gait, LSI Medience Corporation, Japan). Worn for 24 h. DMOs estimated from all WBs.	Stride time.	Multiple linear regression analysis.	No significant association.
Toosizadeh et al. ^20^	MDS-UPDRS part III.	15 Participants, Age: 71.2–(6.3) years, MDS-UPDRS III: 34.8-(10.9) points.	Single wearable device worn in shirt pocket (PAMSys, BioSensics, USA). Worn for 1 day. DMOs estimated from all WBs >3 s.	Walking speed.	Linear regression-ANOVA models and reported as Pearson correlations.	No significant association.

Data presented as mean-(SD), or median-[IQR].

## Discussion

This review is the first to identify how real-world gait DMOs are currently measured and described in PwP, revealing significant variability in measurement protocols and a lack of consensus on optimal data capture methods. DMOs from the pace domain were most characterised, with walking speed being the most widely estimated measure. The majority of DMOs were statistically different between supervised and real-world scenarios, typically reported as ‘lower’ when assessed in the real-world. In comparison to controls, PwP were most consistently reported to walk at slower walking speeds, shorter stride lengths and longer step times with greater variability. DMOs were inconsistently reported to differ between fallers and non-fallers with no study reporting differences for individuals with and without FoG. Differences were observed for individuals with and without fatigue symptoms, and greater motor disability (as measured by H&Y stage). DMOs were only associated with measures of motor severity when estimated from specific WB thresholds or aggregated as extreme values.

In this review, 27 articles were eligible for data extraction, which is substantially fewer than the 307 articles identified in a similar review of DMOs assessed in PwP in a supervised setting^13^. Supervised assessment follows a more well-defined process, where individuals are asked to complete short, scripted tasks which have been extensively implemented within research and clinical settings. They can then be measured by gold standard devices such as instrumented walkways or motion capture systems which have commercial-grade software, enabling simple extraction of DMOs through user-interfaces^40^. Capturing real-world DMOs is more complex, as evidenced in this review, where there is no universally accepted optimal measurement approach. Despite a single wearable worn on the lower back being the most popular device, a wide range of device configurations were reported, each with its own technical limitations in accuracy, reliability and usability^41,42^.

To accurately characterise mobility in daily life, DMOs must be measured over a sufficient time frame to account for day-to-day variability and provide a stable, representative profile of weekly activity. Recent work by Buekers et al.^43^ recommends a minimum of 7 days of continuous monitoring to ensure reliable and valid DMO capture. While the majority of included reports met this 7-day threshold, eight reports recorded for fewer than 3 days, limiting the validity of their findings. Variations in recording duration introduce bias and reduce the comparability of results across reports. Additionally, thirteen reports (33%) did not aggregate data across different WB thresholds. Real-world DMOs are derived from thousands of WBs that differ in length, duration and context. Most WBs are short (<10 s)^17,18,44^ and often capture non-gait-related activities such as transitions, turns, and gait initiation or termination. In contrast, longer WBs (>30 s) better reflect steady-state walking. Consequently, work from the Mobilise-D consortium, suggests that short WBs present the greatest challenge to algorithm performance, suggesting removing short WBs to reduce sources of error and improve DMO accuracy^21,45^. This methodological heterogeneity, combined with the need to analyse large continuous data streams, requires computer programming expertise and the development and validation of bespoke algorithms to extract DMOs. These technical demands may partly explain the limited number of real-world DMO reports and underscore the need for methodological consensus and robust validation protocols to ensure the accuracy, reliability and comparability of real-world DMOs^46^.

Further methodological differences complicate direct report comparisons, particularly in estimating DMOs at the step and stride levels, and estimation of variability. Articles using lower back and foot-based sensors estimated DMOs at the stride level^15,16,19,22,23,28,29,31–33,39^, while those relying solely on lower back-worn devices applied step-based analysis^17,18,21,27,30,34–38^. Stride estimation typically requires a multi-sensor setup, including pressure sensors in the feet for accurate identification of left and right steps. Step-based analysis offers a more granular and straightforward approach, which might be particularly relevant in capturing step to step fluctuations, whereas stride-based analysis provides greater stability by averaging values over the entire gait cycle. Advances in gyroscope-equipped wearable technology and machine-learning techniques now enable robust identification of left and right steps from a single lower-back sensor^47^, suggesting that future reports may transition to stride-based approaches to enhance generalisability across reports.

Additionally, variability in DMOs was analysed using different statistical methods: four reports employed standard deviation (SD)^17,18,27,38^, three used the coefficient of variation (COV)^22,23,29^ and one applied the interquartile range (IQR)^28^. SD provides an absolute measure of variability in original units, making interpretation simpler, whereas COV, allows fairer comparisons across individuals or groups, particularly when means differ. Previous research indicates that both SD and COV exhibit ‘average’ reliability in lab-based settings^48^ and in the real-world are influenced by environmental factors such as indoor versus outdoor walking conditions^49^. Given the complexity of measuring variability in real-world settings, future research should aim to establish the most reliable and stable methodology for capturing gait variability over a 7-day recording period. This is particularly relevant for understanding how motor control is affected in response to fluctuating motor symptoms and medication states in PwP^50,51^.

The majority of DMOs were found to be statistically different between supervised and real-world assessments^15–20^. This highlights how gait differs when assessed in real-world settings in PD, with reports mostly demonstrating slower walking speeds and reduced stride lengths in the real-world compared to supervised assessments. For example, Shah et al.^19^ reported a real-world walking speed of 0.73 m/s, which was significantly lower than the 1.05 m/s recorded during supervised assessment. Two reports of the same study (ICICLE-GAIT) observed slower real-world walking speed (1.01 m/s vs 1.25 m/s) and shorter step length (0.57 m vs 0.66 m), across straight walking^17^ and curvilinear walking^18^, demonstrating how differences exist across a variety of laboratory tasks. Mobility assessment in supervised settings involve structured tasks conducted in controlled environments free from obstructions, changes in lighting and other distractions present in the real-world. Furthermore, supervised assessments are often brief and infrequent, failing to capture the fluctuations in mobility that are common in PD^52,53^. In contrast, real-world settings are uncontrolled, with mobility influenced by factors such as terrain^54^, weather^55^ and different types of indoor and outdoor environments^56^. Additionally real-world mobility influenced by dual task scenarios such as walking while talking or using a smartphone, or by the presence of partners and caregivers. These factors demonstrate the disctinction between real-world and supervised assessments, and how they should be assessed in complement to each-other to deliver a complete profile of mobility in PD.

Only two reports found that DMOs were similar between supervised and real-word assessments. Corrà et al.^16^ found correlations between the 90th percentile of real-world and supervised walking speeds. Another report estimated walking speeds from the upper and lower peaks of a bimodal distribution^15^, finding correlations only between the median walking speed estimated from the fastest, or ‘highest’ modes of the distribution. This suggests that maximal laboratory capacity can reflect real-world maximal capacity and that assessments of maximal capacity are possible within both supervised and real-world environments.

Differences in DMOs between PD and controls were dependent upon the gait domain. DMOs across the pace domain, specifically walking speed, step and stride length, were most consistently reported as different between PD and controls^14,17–26^. DMOs of the pace domain typically capture motor function, particularly in the case of step or stride length, which is a spatial measure of the ability to generate force to move the lower limbs through space ensuring sufficient pacing. These findings align with two previous reviews^13,57^ that only explored DMOs estimated from supervised settings, and demonstrates how impaired muscle contraction, rigidity and postural instability in PD^58^ can negatively affect propulsion, and result in slower walking speed and shorter stride length in both real-world and supervised environments in comparison to older adults. In the rhythm domain, this review found no significant differences in cadence between older adults and PwP^6,21,27,28^. This contrasts with findings from a previous review of supervised DMOs^57^, which reported higher cadence in PD, potentially reflecting a compensatory strategy to maintain walking speed despite shorter stride length^59^. Additionally, the scoping review of supervised DMOs^60^ noted that cadence differed between groups in only 33% of reports (21 out of 65)^13^, suggesting that cadence may not be a consistently marker for distinguishing PD-related gait changes, particularly in real-world settings.

Shah et al.^23^ utilised ROC curve analysis and found that while stride length (AUC = 0.79) and walking speed (AUC = 0.77) approached the discriminatory threshold, only the COV of stride length and swing time demonstrated a discriminant effect (AUC > 0.80). In contrast to spatial-temporal DMOs, turn angle exhibited the highest discriminatory power (AUC > 0.90). Turning is a complex motor action requiring inter-limb coordination, integration of postural and gait function and dynamic control of the centre of mass^60^. Turning deficits in PD arise due to axial rigidity, postural instability and FoG symptoms, leading to varied turning strategies that increase fall risk. Although turning was not included in this review, it represents an important clinical feature in PD. However, there is limited evidence from real-world reports comparing turning characteristics between individuals with PD and controls, highlighting the need for further investigation.

Differences in DMOs across WB thresholds was only explored in three reports which included participants of the ICICLE-GAIT dataset^17,18,21^. Two of these reports included participants from the same time point^17,18^ and found differences in rhythm and asymmetry were dependent upon WB duration only present in medium to long WBs (30–60 s, >60, >120 s). The largest difference in DMOs between PD and control participants at the longest WBs >120 s. Kirk et al.^21^ included participants from a more advanced time point of ICICLE-GAIT and found that walking speed was slower across each WB duration threshold. These findings collectively demonstrate how estimation of DMOs across different WB thresholds enables exploration of how gait is adapted across different real-world contexts. It also demonstrates the importance of clearly explaining which selection of walking bout duration thresholds were included in the report and explain their relevance to the research question.

The reviewed literature compared DMOs across various subgroups, including fallers vs non-fallers. For example, Del Din et al.^27^ reported significant differences in walking speed and step length between 170 respective fallers and non-fallers with PD, which is consistent which previous supervised research^13,61^. In contrast, despite Shah et al.^29^ reporting slower walking speeds in fallers vs non-fallers (0.94 m/s vs 1.05), they found that only turning velocity variability was significantly different between the groups. In high and low risk fallers, Roth et al.^28^ estimated DMOs from ‘level WBs’ (WBs without incline), and only found significant differences with turning characteristics (turning angle variability). In the same dataset, Ullrich et al.^31^ did not control for level walking, and reported reduced walking speed and stride length as distinguishing factors between high and low risk fallers. Direct comparison between these reports is limited as they all selected different approaches for measurement devices, WB thresholds and step or stride-based analysis. Approaches to quantify gait and it’s relation to falls are based upon assessment over multiple supervised tasks, reduced step length is the primary determinant of minimum toe clearance which increases the risk of falls^62^. For example, Polhemus et al.^13^ reported that step and stride lengths were associated with falls in 10/10 (100%) of the reports and walking speed in 14/18 reports (77.8%). In contrast, Haertner et al.^63^ demonstrated that turning characteristics assessed in supervised and real-world settings were significantly associated with only fear of falling rather than positive falls history. Given this contrasting evidence, this warrants a larger investigation of faller status and falls risk which includes both spatial-temporal and turning DMOs estimated with the same protocol.

Three reports explored differences between individuals with and without FoG^32–34^, with all reports reporting no significant differences in walking speed and step time between the groups. FoG is speculated to be contextually sensitive^64^, and may occur during shorter WBs in confined, home-based environments that involve sharp turns^33^. Interestingly, Mancini et al.^33^ found that turning characteristics could effectively distinguish freezers from non-freezers. As FoG causes an episodic inability to generate effective stepping^65^, DMO algorithms may inadvertently exclude steps and gait sequences where FoG occurs, demonstrating the need for careful selection of thresholds to exclude initial contact events, when assessing someone that experiences FoG symptoms. Aside from a previous fall, FoG is one of the most prominent risk factors for increased fall risk in PD, who are at greater risk of falls in comparison to older adults without PD^66^. Clinically FoG is assessed through the FoG questionnaire which evaluates the self-reported frequency, severity, duration and impact of FoG symptoms^64^. Real-world DMOs could complement the existing assessment, to provide a more ecologically valid, data driven approach to understanding how self-reported FoG symptoms truly influence someone’s functional mobility in their daily life.

One report explored the influence of fatigue symptoms upon real-world mobility in PD^35^, finding that PwP with fatigue have greater difficulty regulating their timing and rhythm of gait activity, which was reflected by significantly higher step time, step time variability and step time asymmetry in those with fatigue. These gait disturbances were greater during long WBs, demonstrating how prolonged walking is more challenging for people with fatigue symptoms^35^. This inability to regulate gait could increase risk of falling and sustaining a serious injury or lead to individuals limiting their out of home mobility, which increases negative effects of physical inactivity^67^ and may reduce independence and quality of life^10^. Further reports exploring the real-world impact of fatigue symptoms with DMOs need to be completed before conclusions can be drawn.

Three reports reported significant associations between DMOs and the MDS-UPDRS III^21,38,39^, in contrast to the 87 reports of associations found with supervised DMOs in PD^13^. The MDS-UPDRS III is a comprehensive measure capturing a wide range of motor signs, including upper limb function and tremor, assessed during a brief clinical visit. Many of the supervised DMOs were estimated from straight walking assessments, which is similar to the gait-item of the MDS-UPDRS III. Notably, two reports that identified associations with the MDS-UPDRS III utilised specific aggregation thresholds. Kirk et al.^21^ reported a significant but weak negative association between walking speed and WBs between 30 and 60 s. Corrà et al.^16^ investigated associations with the MDS-UPDRS III gait item, finding significant relationships with the 90th percentile of walking speed when participants were ON medication and the 25th percentile when OFF medication. Two additional reports identified associations between pace DMOs and the PIGD score^23,39^. This is logical, as the PIGD score is calculated using the gait-related items from the MDS-UPDRS III^6^ in addition to items from MDS UPDRS part II. Overall, the weak to moderate relationships identified across reports with components of the MDS-UPDRS III is not surprising given the diverse range of motor signs, including upper limb and tremor, assessed compared to gait measurements over 7-days. The weak to moderate relationships identified between DMOs and components of the MDS-UPDRS III are not surprising, given that the MDS-UPDRS III score and real-world DMOs assess different aspects of mobility. Thus, moderate to weak correlations indicate that DMOs have some sensitivity to clinical assessments but also capture unique information about real-world mobility performance that may not be observable during short clinical assessments. Therefore, real-world DMOs could measure unique aspects of mobility that can be applied to complement the existing clinical assessment of motor severity.

Mirelman et al.^38^ employed machine learning feature selection and aggregated DMOs across all WBs greater than 30 s. They identified step length as the strongest independent feature that most accurately predicted MDS-UPDRS III scores. While the MDS-UPDRS III includes a gait item that objectively measures step length, it constitutes only a small fraction of the overall score (4 out of 132 points)^6^. Therefore, it is unsurprising that step length shows the strongest association with clinical measures of motor severity compared to other DMOs. Interestingly, a larger set of features not included in this review, such as relative amplitude, non-active gait time and mean signal vector magnitude, were ranked among the top 15 features. While spatial-temporal DMOs provide valuable insights into how motor symptoms affect functional mobility, these novel features may offer superior classification accuracy for early PD diagnosis and objective monitoring of disease progression^68^. Data-driven machine learning approaches like this are critical for determining the most relevant characteristics, suggesting that using DMOs in isolation may be less effective. Instead, combining multiple variables within specific domains in composite models could enhance their application, improving the classification of PD and providing more objective markers for motor severity.

One report explored changes across Hoehn and Yahr Stages^25^, finding that walking speed significantly decreased whilst stride time became significantly faster in PD patients in the modified Hoehn and Yahr stage 1.5 compared to those in stages 2.5–3.0. Hoehn and Yahr stage is a five-point item that is part of the MDS-UPDRS III, where the clinician rates motor disability from 0 (no impairment) to 5 (severely impaired, wheelchair bound). While this was the only report to explore differences across Hoehn and Yahr stages, it only assessed participants over 25 h, meaning it would be expected that any new report addressing a similar research question would find gait impairment to be stronger as Hoehn and Yahr stages increases.

The review process faced several limitations. A meta-analysis was not feasible due to heterogeneity in measurement protocols across reports, limiting the ability to synthesise findings quantitatively. Publication bias may also have influenced the results, as reports reporting significant effects are more likely to be published, potentially skewing the overall conclusions. Furthermore, the review was conducted in two stages, involving five researchers at different phases. Ideally, a single-phase review with a consistent group of reviewers would have ensured greater methodological consistency.

As the field of literature grows, researchers should adopt protocols which build on the expert consensus for capturing real-world DMOs that has been outlined in the lessons learnt by the Mobilise-D consortium^45,46,69,70^. There are a limited number of reports that have characterised DMOs across multiple time points in PD to understand their sensitivity to measuring progression, and whether changes in DMOs can predict changes in clinical outcomes. There are a limited number of studies that have characterised DMOs across multiple timepoints in PD to understand their sensitivity to measuring progression, and whether changes in DMOs can predict changes in clinical outcomes. This may be the result of technical challenges which are now being addressed, such as understanding the optimal measurement protocol (ie., device configuration and length of assessment) and the best algorithms for extracting DMOs from large data streams with clinically acceptable accuracy, precision and reliability, which limits the confidence of researchers to collect longitudinal data. This is being addressed by consortiums such as Mobilise-D in their clinical validation study which has assessed DMOs in a large population of people with PD, with results soon to be published^71^. Furthermore, there is limited consensus on what the best statistical approach is toward modelling the complex interplay between real-world DMOs and clinical outcomes. In order to understand whether DMOs can predict important clinical outcomes, such as development of specific symptoms or changes in medication use, this is an essential gap that must be addressed in the literature^9,11,13^.

The review identified 17 reports between 2000–2021 and 10 reports between 2021–2024, which demonstrates the rapidly growing field of real-world literature. A limitation of this work is that an up-to-date screening has not occurred; however, since the search of this review (April 2024) was run until the present day (2025), an additional 757 reports prior to duplicate removal and abstract screening have been identified. Select examples of articles that would be included in this review are the work of Nishi et al.^72^, who addressed objectives two, four and five, finding walking speed to be significantly lower in the real world compared to supervised assessments. Furthermore, they expanded upon the bimodal approach proposed by Atrasaei et al.^15^, and found associations between the fit of the distribution and motor severity and quality of life. Additionally, DMOs have been implemented as secondary outcome measures in a large multicentre phase 3 clinical trial^73^, with results yet to be published.

Eleven reports included participants from the same studies, with participants having a relatively similar range in age and motor impairment. Therefore, there is a need for future studies to include individuals with prodromal PD, those recently diagnosed, or those with more advanced motor impairment. This review focused on spatial-temporal gait DMOs, which capture real-world functional capabilities. Future reviews could expand on this by including turning DMOs, which may have particular clinical relevance in the context of falls and FoG events^29,32^. Additionally, researchers have begun to quantify more complex DMOs or model multiple DMOs within domains using machine learning, deep learning and signal processing techniques applied to real-world datasets^68,74^ alongside the work by Mirelman et al.^38^, which aims to reduce a large number of features into the most clinically relevant measures. A promising future research direction in PD is to investigate whether more complex DMOs or composite models offer stronger clinical validity, particularly for early diagnosis and objective measurement of motor severity. However, spatial-temporal DMOs remain advantageous in terms of interpretability for clinicians and patients.

Real-world DMOs have potential utility to inform future research and clinical practice. This review has demonstrated that supervised and real-world assessments evaluate different aspects of mobility in PD, thus it can be argued that use of either assessment in isolation does not present the complete picture of mobility impairments experienced by PwP. Real-world functional mobility is more impaired in PD, who typically walk with slower walking speed and reduced stride length, in comparison to matched controls. Thus, real-world DMOs could be used to complement the existing supervised assessment of motor severity in PD. From a clinical perspective, it is essential to understand how novel signal-based characteristics and machine learning models can enhance the current clinical diagnosis and management of PD. As the field progresses in terms of technologies and algorithms, DMOs can be characterised with increasing accuracy and reliability. Accordingly, there is a need to consistently evaluate their construct validity and their ability to measure longitudinal changes in response to PD progression.

## Methods

### Review methodology

This systematic review of real-world DMOs adopted a similar approach to a large review that was conducted as part of the Mobilise-D project^75^, that only included DMOs assessed in a supervised setting. This review was registered on PROSPERO (CRD42021281213) and adhered to the PRISMA guidelines^76^. The only changes to the protocol were a revised title and author list. A separate protocol manuscript was not prepared.

### Search strategy

Systematic searches were iteratively developed across seven databases for peer-reviewed literature: MEDLINE, EMBASE, CINAHL, Scopus, Web of Science, IEEE Xplore and ACM Digital Library. The search strategy was tested with a research librarian and subject matter experts using medical subject headings (MeSH) and free-text terms based on the key concepts of gait analysis, wearable sensors and PD. The search included variations of spatiotemporal gait parameters, real-world mobility assessment and PD-specific terminology. This search was conducted in two phases, the initial review was limited to reports from January 2000 to October 2021, with a follow up search conducted from October 2021 to April 2024. Full details of the search strategy implemented across each database is provided in Supplementary Tables 1–5.

### Study selection

Full eligibility criteria of articles can be found in (Table 6).

**Table 6:** Inclusion and exclusion criteria of this systematic review

	Inclusion Criteria	Exclusion Criteria
Language	• Published in English language.	• Published in a language other than English.
Time frame	• Published before April 2024 (Search start date of the review).	• Any report that was published prior to 1999.
Location/Setting	• Real-world/home setting. • Laboratory/clinic setting will only be included if it has been included alongside real-world assessment in the same report.	• Real-world/home assessment which is supervised and scripted and supervised. • A report that has assessed DMOs only during scripted laboratory testing.
Topic	• Reports that have quantitatively measured DMOs in PD, in accordance with Objectives two, three, four and five.	• Reports that have only quantified non-included DMOs. • Reports that did not answer one of the four objectives. • Qualitative data.
Population	• Adults with a clinical diagnosis of PD. • Matched control participants will only be included alongside a cohort of PwP addressing objective two.	• Children. • Adults without a diagnosis of PD.
Publication Type	• Peer reviewed publications. • Grey literature.	• Conference abstracts. • Posters. • Study protocols. • Reviews. • Meta-analyses. • Interventional studies. • Case reports.

DMOs could include any digital measures encompassed by the ICF definition of ‘mobility.’ However, our scope is limited to a set of 23 gait-related DMOs in accordance with the primary objective of Mobilise-D (Table 7). This list was compiled in consultation with mobility experts, technologists and clinicians and includes spatiotemporal parameters categorised into the domains of the gait model; namely pace, rhythm, variability, asymmetry and postural control^13,77^. This conceptual model was adapted to include cadence, and stride-based DMOs, while step width variability, which cannot be calculated from wearable data, is excluded.

**Table 7:** Spatial-temporal DMOs included in this review and organised in accordance with the conceptual gait model of Lord et al.^77,79^

Domain	DMO
Pace	• Step length. • Stride length. • Swing time variability. • Walking speed.
Rhythm	• Cadence. • Double support time. • Single support time. • Stance time. • Step time. • Stride time. • Swing time.
Variability	• Cadence variability. • Stance time variability. • Step time variability. • Step length variability. • Stride length variability. • Stride time variability. • Walking speed variability.
Asymmetry	• Stance time asymmetry. • Step time asymmetry. • Stride time asymmetry. • Swing time asymmetry.
Postural control	• Step length asymmetry.

This review focused on gait-related DMOs due to their clinical interpretability, technical maturity and relevance to real-world walking in PD. These outcomes are based upon validated biomechanical frameworks and are feasible to implement using current wearable technology. More complex signal-based features and composite models have great promise but require further standardisation of core variables and validation for long-term use in unsupervised real-world settings. Similarly, a consistent definition of core turning metrics with clear validation data will provide greater clarity in the future for additional variable selection. As the field evolves, we anticipate future reviews will be needed to assess these emerging measures.

For objective 5, only reports that measured motor severity with the components of the MDS-UPDRS III^6^, such as part III total score, the MDS-UPDRS III-gait item and the postural instability gait disorder (PIGD) score are included. Motor disability was assessed with Hoehn and Yahr stage.

Articles were excluded following a hierarchical approach: (1) absence of an available abstract; (2) ineligible study type; (3) no human participants; (4) no participants with PD; (5) absence of any included DMO; (6) lack of real-world participant assessment; (7) failure to address a relevant research question.

Eligibility of articles was assessed through abstract and full text screening. All reviewers (CK, EP, MM, HB and SS) received training on review conduct prior to abstract screening and piloted eligibility criteria on a random set of 50 abstracts to ensure consistency. Four reviewers independently screened the abstracts, where the lead reviewer (CK) monitored consistency between reviewers throughout the abstract screening stage. Articles were included in the full-text screening if a single reviewer deemed an abstract eligible; articles were excluded if two reviewers opted for rejection. For the abstract screening, study design, review conduct, records of duplication, reference exclusion on an individual author contribution were managed in Rayyan (Cambridge, Massachusetts, USA). Initially, references were compiled in Zotero (George Mason University, Virginia, USA) and the final review libraries in Mendeley (Elsevier B.V., Amsterdam, The Netherlands). Any duplicate reports were excluded, based upon comparing titles, authors, publication years and abstracts. Two reviewers; CK, MM in the original review and CK, EP in the review update, screened each full-text and extracted data if eligible, where all the data extraction items from the review can also be viewed in Supplementary Table 11. Following data-extraction, disagreements were resolved through discussion. Data extraction forms were developed, and data extraction was conducted in Microsoft Excel (Microsoft, Washington, USA).

### Analysis of eligible articles

The number of eligible articles was recorded for each study objective. For each objective, clinical and demographic data were systematically extracted as reported by the original authors. These data were then summarised using either: (i) a pooled mean, weighted by sample size to account for differences in study populations; or (ii) a median of medians approach for reports that used median values.

A customised quality appraisal tool was developed based upon the Quality Assessment Tool for observational cohort and cross-sectional studies^78^, which was assessed by three reviewers (CK, MM and EP). This assessed risk of bias in included reports, tailored to DMOs. The tool addressed selection bias by evaluating whether report populations and inclusion/exclusion criteria were clearly defined; information bias through assessment of protocol transparency, device reporting and data collection consistency; and reporting bias by examining statistical appropriateness, probability value reporting, confounder adjustment and selective outcome reporting. Due to heterogeneity of reports, the quality assessment was customised to allow evaluation of aspects of measurement approach that could affect the internal validity of continuous measurement of real-world DMOs. For example, sensor attachment, length of real-world recording and measurement devices can all influence validity. The complete list of items in the quality appraisal can be found in Supplementary Table 12. The classification of each quality score is described in ref. ^78^.

Report findings were synthesised narratively due to heterogeneity in report designs, populations, digital DMO definitions and assessment settings. Extracted data were grouped according to pre-defined research questions and DMO domains (e.g. pace, rhythm, variability). For each group, we summarised whether statistically significant differences or associations were reported, based on the original report analyses. When reports contributed to multiple research questions or outcomes, they were considered in each relevant category. The synthesis followed PRISMA 2020 guidelines, with results presented in summary tables to transparently display the strength and direction of findings across reports.

Completed PRIMSA 2020 checklist can be found in Supplementary Table 13.

## Supplementary information

Supplementary materials

## Data Availability

The datasets of this review are available from the corresponding author on reasonable request.
