# Supplementary material for: A systematic review of real-world gait-related digital mobility outcomes in Parkinson’s disease
Source: NPJ Digit Med. 2025 Sep 30;8:585. doi: 10.1038/s41746-025-01938-y (PMC12484654; doi:10.1038/s41746-025-01938-y)
Supplement: Supplementary file 1 — Supplementary materials [file 41746_2025_1938_MOESM1_ESM.pdf]

Supplementary materials: **A Systematic Review of Real-World Gait-Related Digital Mobility Outcomes in Parkinson's Disease**

Table of contents:

Supplementary Figure 1. Individual exclusion reasons from the abstract screening……..…2
Supplementary Table 1. List of reports that were excluded during the full text review, alongside their exclusion reasons..3
Supplementary Table 2. Quality assessment reported across each individual study included in this review addressing objective two (real-world vs supervised assessment)......................8
Supplementary Table 3. Quality assessment reported across each individual study included in this review addressing objective three (People with PD compared to controls without PD). 9
Supplementary Table 4. Quality assessment reported across each individual study included in this review addressing objective four (Comparisons across PD sub-groups)...................11
Supplementary Table 5. Quality assessment reported across each individual study included in this review addressing objective five (associations with motor severity)........................13
Supplementary Table 6. Differences identified in the measurement methods of the supervised studies...15
Supplementary Table 7. EMBASE and MEDLINE Search strategy applied in the systematic review...16
Supplementary Table 8. CINHL Search strategy applied in the systematic review............17
Supplementary Table 9. CINAHL Search strategy applied in the systematic review..........18
Supplementary Table 10. Web of Science search strategy applied in the systematic review. 19
Supplementary Table 11. IEEE explore search strategy applied in the systematic review...19
Supplementary Table 12. Table of data extraction methods for the full-text review..........20
Supplementary Table 13. Quality appraisal assessment...22
Supplementary Table 14. Completed PRIMSA checklist of this systematic review...........23

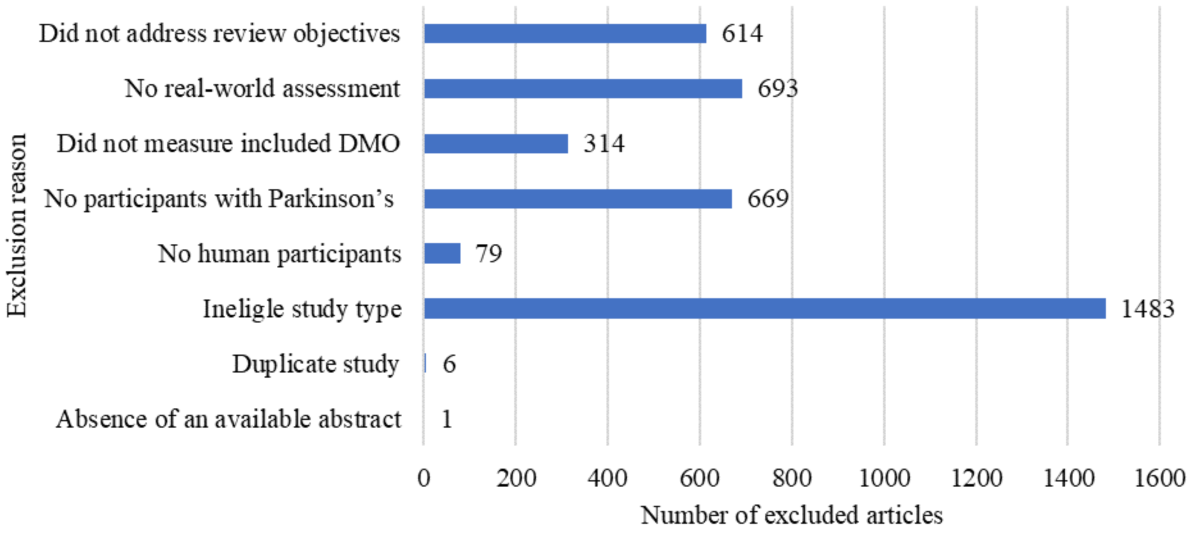

Supplementary Figure 1. Individual exclusion reasons from the abstract screening.

Supplementary table 1. List of reports that were excluded during the full text review, alongside their exclusion reasons.

**Study**	**Exclusion reason**
Abujrida et al. 2022[1]	Absence of any included DMO
Aich et al. 2020[2]	Absence of any included DMO
Alazeb et al. 2024[3]	Absence of any included DMO
Atrsaei et al. 2021[4]	Duplicate report
Atrsaei et al. 2021[5]	Failure to address a relevant research question
Balakrishnan et al. 2023[6]	Lack of real-world participant assessment
Barry et al. 2014[7]	Ineligible study type (conference abstract)
Beigi et al. 2022[8]	Absence of any included DMO
Bernad-Elazari et al. 2016[9]	Absence of any included DMO
Bernad-Elazari et al. 2016[9]	Duplicate report
Bianchini et al. 2022[10]	Lack of real-world participant assessment
Bianchini et al. 2023[11]	Absence of any included DMO
Bobic et al. 2023[12]	Absence of any included DMO
Borzi et al. 2023[13]	Absence of any included DMO
Brand et al. 2022[14]	Absence of any included DMO
Brink-Kjaer et al. 2023[15]	Full text unavailable
Burq et al. 2022[16]	Absence of any included DMO
Busse et al. 2004[17]	Absence of any included DMO
Caballol, et al. 2023[18]	Failure to address a research question
Cai et al. 2023[19]	Lack of real-world participant assessment
Cesarelli et al. 2022[20]	Lack of real-world participant assessment
Chen et al 2020[21]	Absence of any included DMO
Chen et al 2020[21]	Duplicate report
Cheng et al. 2017[22]	Absence of any included DMO
Cheng et al. 2018[23]	Ineligible study type (conference abstract)
Cherukuri, et al. 2022[24]	Lack of real-world participant assessment
Cohen et al. 2023[25]	Failure to a relevant research question.
Conradsson, et al. 2024[26]	Absence of any included DMO

Cook et al. 2015[27]	Absence of any included DMO
Cook et al. 2015[27]	Duplicate report
D'Arco et al. 2022[28]	Absence of any included DMO
da Rosa Tavares et al. 2023[29]	Absence of any included DMO
de Faria J. and Sousa et al. 2023[30]	Lack of real-world participant assessment
Del Din et al. 2020[31]	No participants with Parkinson's disease
Dinesh et al. 2022[32]	Absence of any included DMO
Divyashree and Dwivedi. 2023[33]	Absence of any included DMO
Domingues et al. 2022[34]	Lack of real-world participant assessment
Elavaar Kuzhali et al. 2023[35]	Absence of any included DMO
El-Gohary et al. 2014[36]	Absence of any included DMO
Escamilla-Luna et al. 2022[37]	Failure to address a research question
Galperin et al.[38]	Absence of any included DMO
Galperin et al.[38]	Duplicate report
Ganesh et al. 2023[39]	Absence of any included DMO
Ginis et al. 2023[40]	Absence of any included DMO
Goh et al. 2022[41]	Absence of any included DMO
Gong et al. 2023[42]	Ineligible study type (pre-print)
Goni et al. 2022[43]	Lack of real-world participant assessment
Hao et al. 2023[44]	Absence of any included DMO
Haruyama et al. 2021[45]	Lack of real-world participant assessment
Hausdorff et al. 2007[46]	Ineligible study type (conference abstract)
Hausdorff et al. 2007[46]	Duplicate report
Herman et al. 2014[47]	Lack of real-world participant assessment
Ho et al. 2024[48]	Lack of real-world participant assessment
Hobert et al. 2019[49]	Lack of real-world participant assessment
Holleran et al. 2020[50]	Absence of any included DMO
Horak et al. 2018[51]	Ineligible study type (conference abstract)
Ingelse et al. 2022[52]	Absence of any included DMO
Jiang et al. 2024[53]	Lack of real-world participant assessment
Kataoka H. and Sugie K. 2021[54]	Absence of any included DMO

Kegelmeyer et al. 2024[55]	Lack of real-world participant assessment
Kirk et al. 2024[56]	Lack of real-world participant assessment
Laurent et al. 2023[57]	Lack of real-world participant assessment
Leavy et al. 2018[58]	Lack of real-world participant assessment
Leavy et al. 2023[59]	Absence of any included DMO
Li et al. 2022[60]	Absence of any included DMO
Li et al. 2022[61]	Lack of real-world participant assessment
Li et al. 2023[62]	Absence of any included DMO
Lin et al. 2022[63]	Absence of any included DMO
Liu et al. 2022[64]	Lack of real-world participant assessment
Lopez-Delgado et al. 2023[65]	Absence of any included DMO
Lord et al. 2013[66]	Absence of any included DMO
Mactier et al. 2015[67]	Absence of any included DMO
Malutan et al. 2022[68]	Absence of any included DMO
Mancini et al. 2018[69]	Ineligible study type (conference abstract)
Mancini et al. 2023[70]	Lack of real-world participant assessment
Mc Ardle et al. 2022[71]	Failure to address a research question
Mercuri et al. 2022[72]	Lack of real-world participant assessment
Mitoma et al. 2010[73]	Failure to address a research question
Mitoma et al. 2010[73]	Duplicate report
Moon et al. 2021[74]	Full-text unavailable
Moradi et al. 2022[75]	Absence of any included DMO
Moradi et al. 2023[76]	Failure to address a research question
Morgan et al. 2023[77]	Ineligible study type (dataset)
Morris et al. 2017[78]	Failure to address a research question
Morris et al. 2019[79]	Ineligible study type (conference abstract)
Naimi et al. 2023[80]	Absence of any included DMO
Negi et al. 2022[81]	Absence of any included DMO
Nouriani et al. 2023[82]	Absence of any included DMO
Olmos and Martinez. 2022[83]	Lack of real-world participant assessment
Otlet and Ronsse. 2022[84]	Absence of any included DMO

Oyama et al. 2023[85]	Lack of real-world participant assessment
Pallavi et al. 2023[86]	Lack of real-world participant assessment
Patil et al. 2022[87]	Absence of any included DMO
Pelicioni et al. 2023[88]	Lack of real-world participant assessment
Pham. 2022[89]	Absence of any included DMO
Pisal et al. 2018[90]	Failure to address a research question
Porta et al. 2018[91]	Absence of any included DMO
Porta et al. 2018[91]	Duplicate report
Prusynski et al. 2022[92]	Absence of any included DMO
Pullagura et al. 2022[93]	Absence of any included DMO
Rabuffetti et al. 2023[94]	Absence of any included DMO
Rastegari et al. 2017[95]	Lack of real-world participant assessment
Rastegari et al. 2022[96]	Absence of any included DMO
Raykov et al. 2021[97]	Absence of any included DMO
Raykov et al. 2021[97]	Absence of any included DMO
Rochester et al. 2012[98]	Failure to address a research question
Roth et al. 2021[99]	Lack of real-world participant assessment
Shah et al. 2019[100]	Ineligible study type (conference abstract)
Shah et al. 2020[101]	Absence of any included DMO
Shah et al. 2020[102]	Ineligible study type (conference abstract)
Shih et al. 2022[103]	Ineligible study type (Pre-print)
Shih et al. 2023[104]	Absence of any included DMO
Shimada et al. 2021[105]	No participants with Parkinson’s disease
Shokouhi et al. 2022[106]	Absence of any included DMO
Silva de Lima et al. 2018[107]	Absence of any included DMO
Silva et al. 2021[108]	Lack of real-world participant assessment
Singh, et al. 2023[109]	Absence of any included DMO
Sorici et al. 2023[110]	Ineligible study type (protocol)
Stuckenschneider et al. 2021[111]	Absence of any included DMO
Sturchio et al. 2021[112]	Lack of real-world participant assessment
Sütçü et al. 2021[113]	Full-text unavailable

Terashi et al. 2019[114]	Ineligible study type (conference abstract)
Terashi et al. 2022[115]	Absence of any included DMO
Terashi, et al. 2012[116]	Absence of any included DMO
Tian et al. 2024[117]	Absence of any included DMO
Tigrini et al. 2023[118]	Absence of any included DMO
Tsakanikas et al. 2021[119]	Lack of real-world participant assessment
Tucak et al. 2023[120]	Absence of any included DMO
Ullrich et al. 2021[121]	Failure to address a research question
Utsumi et al. 2012[122]	Failure to address a research question
Utsumi et al. 2012[123]	Failure to address a research question
Vajiha Begum et al. 2022[124]	Absence of any included DMO
Vila et al. 2021[125]	Lack of real-world participant assessment
von Der Recke et al. 2023[126]	Lack of real-world participant assessment
von Rosen et al. 2021[127]	Lack of real-world participant assessment
Waddell et al. 2023[128]	Failure to address a research question
Wells et al. 2023[129]	Ineligible study type (protocol)
Williamson et al. 2021[130]	Absence of any included DMO
Winkler et al. 2022[131]	Lack of real-world participant assessment
Wu et al. 2022[132]	Lack of real-world participant assessment
Yang et al. 2022[133]	Absence of any included DMO
Yogev-Seligmann et al. 2023[134]	Lack of real-world participant assessment
Yokote et al. 2022[135]	Lack of real-world participant assessment
Yoneyama et al. 2013[136]	Absence of any included DMO
Yoneyama et al. 2016[137]	Absence of any included DMO
Yue et al. 2024[138]	Absence of any included DMO
Zajac et al. 2022[139]	Failure to address a research question
Zeng e al. 2023[140]	No participants with Parkinson's disease
Zeng et al. 2023[141]	Absence of any included DMO
Zhao et al. 2021[142]	Lack of real-world participant assessment
Zhao et al. 2023[143]	Lack of real-world participant assessment
Zoetewei et al. 2024[144]	Absence of any included DMO

Supplementary Table 2. Quality assessment reported across each individual study included in this review addressing objective two (real-world vs supervised assessment).

Study		Population clearly defined?	Inclusion and exclusion criteria defined?	Study objective clearly stated?	Main outcomes clearly described?	Validated measures implemented consistently?	Mesaurement device clearly reported?	Protocol clearly described?	Sensor attachment reported?	Length of real-world assessment clearly described?	> 3 days of collected data controlled for?	Appropriate ethics and consent?	Were the statistical tests appropriate?	Probability values reported for the main outcomes?	Confounding variables statistically adjusted?	Reporting of results adequate	Average score
Atrsaei, et al., 2021	R 1	Y	Y	Y	Y	Y	Y	Y	N	Y	N	Y	Y	Y	Y	Y	13
	R 2	Y	Y	Y	Y	Y	Y	Y	N	Y	N	Y	Y	Y	Y	Y	12
Corra, et al., 2021	R 1	Y	Y	Y	Y	Y	Y	Y	N	N	N	Y	Y	Y	Y	Y	12
	R 2	Y	Y	Y	Y	Y	Y	Y	N	Y	N	Y	Y	N	N	Y	11
Del Din, et al., 2016	R 1	Y	Y	Y	Y	Y	Y	Y	Y	Y	N	Y	Y	Y	N	N	12
	R 2	Y	Y	Y	Y	Y	Y	Y	Y	Y	Y	Y	Y	N	Y	Y	13
Rehman, et al., 2022	R 1	Y	Y	Y	Y	Y	Y	Y	N	Y	N	Y	Y	Y	Y	Y	13
	R 2	Y	Y	Y	Y	Y	Y	Y	N	Y	N	Y	Y	Y	Y	Y	13
Shah, et al., 2020c	R 1	Y	Y	Y	N	Y	Y	N	N	N	N	Y	Y	Y	Y	Y	8
	R 2	Y	Y	Y	Y	Y	Y	Y	N	Y	Y	Y	N	N	N	N	10
Toosizadeh, et al., 2015	R 1	Y	Y	Y	N	Y	N	N	Y	Y	N	Y	Y	N	N	Y	9
	R 2	Y	Y	Y	Y	Y	N	N	Y	Y	N	Y	Y	N	N	Y	10

'R' denotes reviewer

Supplementary Table 3. Quality assessment reported across each individual study included in this review addressing objective three (People with PD compared to controls without PD).

Study		Population clearly defined?	Inclusion and exclusion criteria defined?	Study objective clearly stated?	Main outcomes clearly described?	Validated measures implemented consistently?	Mesaurement device clearly reported?	Protocol clearly described?	Sensor attachment reported?	Length of real-world assessment clearly described?	> 3 days of collected data controlled for?	Appropriate ethics and consent?	Were the statistical tests appropriate?	Probability values reported for the main outcomes?	Confounding variables statistically adjusted?	Reporting of results adequate	Average score
Adams, et al., 2021	R1	Y	N	Y	Y	N	Y	Y	N	Y	N	Y	Y	Y	N	Y	10
	R2	Y	N	Y	Y	N	Y	Y	N	Y	N	Y	Y	Y	N	Y	10
Adams, et al., 2023	R1	Y	Y	Y	N	N	N	N	N	N	Y	Y	Y	Y	Y	N	8
	R2	Y	Y	Y	N	N	N	N	N	N	Y	Y	Y	Y	Y	N	8
Del Din, et al., 2016	R1	Y	Y	Y	Y	Y	Y	Y	Y	Y	N	Y	Y	Y	N	N	12
	R2	Y	Y	Y	Y	Y	Y	Y	Y	Y	Y	Y	Y	N	Y	Y	13
Kirk, et al., 2023	R1	Y	Y	Y	Y	Y	Y	Y	Y	Y	Y	Y	Y	Y	Y	Y	15
	R2	Y	Y	Y	Y	Y	Y	Y	Y	Y	Y	Y	Y	Y	Y	Y	15
Rehman, et al., 2022	R1	Y	Y	Y	Y	Y	Y	Y	N	Y	N	Y	Y	Y	Y	Y	13
	R2	Y	Y	Y	Y	Y	Y	Y	N	Y	N	Y	Y	Y	Y	Y	13
Shah, et al., 2020a	R1	Y	Y	Y	Y	Y	Y	Y	N	Y	N	Y	Y	Y	N	Y	12
	R2	Y	Y	Y	Y	Y	Y	Y	Y	Y	Y	Y	Y	Y	Y	Y	15
Shah, et al., 2020b	R1	N	Y	Y	Y	Y	N	N	N	N	N	Y	Y	Y	Y	N	9
	R2	Y	Y	Y	Y	Y	Y	Y	N	Y	Y	Y	Y	Y	0	N	12
Shah, et al., 2020c	R1	Y	Y	Y	N	Y	Y	N	N	N	N	Y	Y	Y	Y	Y	8
	R2	Y	Y	Y	Y	Y	Y	Y	N	Y	Y	Y	N	N	N	N	10
Terashi, et	R1	Y	Y	Y	Y	N	Y	Y	Y	Y	N	Y	Y	Y	Y	Y	12

al., 2020	R 2	Y	Y	Y	Y	Y	Y	Y	Y	Y	N	Y	N	N.5	Y	Y	12
Terashi, et al., 2013	R 1	Y	N	Y	Y	N	Y	Y	Y	Y	N	N	N	N	N	Y	9
	R 2	Y	Y	Y	Y	N	Y	N	N	Y	N	Y	N	N	N	Y	8

'R' denotes reviewer

Supplementary Table 4. Quality assessment reported across each individual study included in this review addressing objective four (Comparisons across PD sub-groups)

Study		Population clearly defined?	Inclusion and exclusion criteria defined?	Study objective clearly stated?	Main outcomes clearly described?	Validated measures implemented consistently?	Mesaurement device clearly reported?	Protocol clearly described?	Sensor attachment reported?	Length of real-world assessment clearly described?	> 3 days of collected data controlled for?	Appropriate ethics and consent?	Were the statistical tests appropriate?	Probability values reported for the main outcomes?	Confounding variables statistically adjusted?	Reporting of results adequate	Average score
Del Din, et al., 2019	R 1	Y	Y	Y	Y	Y	N	Y	N	Y	N	Y	Y	N	Y	Y	11
Del Din, et al., 2019	R 2	Y	Y	Y	Y	Y	Y	Y	Y	Y	Y	Y	Y	N	Y	N	13
Mancini, et al., 2018	R 1	N	Y	Y	N	N	Y	Y	Y	Y	N	N	N	Y	N	Y	8
Mancini, et al., 2018	R 2	N	Y	Y	N	Y	Y	Y	Y	Y	N	N	N	Y	N	Y	9
Mancini, et al., 2021	R 1	Y	Y	Y	Y	Y	Y	Y	N	Y	N	Y	Y	Y	Y	Y	13
Mancini, et al., 2021	R 2	Y	Y	Y	Y	Y	Y	Y	N	Y	Y	Y	0	Y	Y	Y	13
Pilotto, et al., 2023	R 1	Y	Y	Y	Y	Y	N	Y	N	Y	N	Y	Y	Y	Y	Y	12
Pilotto, et al., 2023	R 2	Y	Y	Y	Y	Y	N	Y	N	Y	N	Y	Y	Y	N	Y	11
Roth, et al., 2022	R 1	Y	N	Y	Y	Y	Y	Y	Y	Y	N	Y	Y	Y	N	Y	12
Roth, et al., 2022	R 2	Y	N	Y	Y	Y	Y	Y	Y	Y	N	Y	Y	Y	N	Y	12
Ullric, et al., 2023	R 1	Y	Y	Y	Y	Y	Y	Y	Y	Y	Y	Y	Y	Y	Y	Y	15
Ullric, et al., 2023	R 2	Y	Y	Y	Y	Y	Y	Y	Y	Y	Y	Y	Y	Y	Y	Y	15
Weiss, et al., 2015	R 1	Y	Y	Y	Y	Y	N	Y	Y	Y	N	Y	N	Y	N	Y	11
Weiss, et al., 2015	R 2	Y	Y	Y	Y	Y	Y	Y	Y	Y	N	Y	Y	Y	N	Y	13
Weiss, et al., 2014	R 1	Y	Y	Y	Y	Y	N	Y	Y	Y	N	Y	N	Y	N	Y	11
Weiss, et al., 2014	R 2	Y	Y	Y	Y	Y	Y	Y	Y	Y	N	Y	Y	Y	N	Y	13
Weiss, et al., 2015	R 1	Y	Y	Y	Y	Y	Y	Y	Y	Y	N	Y	Y	Y	N	Y	13
Weiss, et al., 2015	R 2	Y	Y	Y	Y	Y	Y	Y	Y	Y	N	Y	Y	Y	Y	Y	14

‘R’ denotes reviewer

Supplementary Table 5. Quality assessment reported across each individual study included in this review addressing objective five (associations with motor severity).

Study		Population clearly defined?	Inclusion and exclusion criteria defined?	Study objective clearly stated?	Main outcomes clearly described?	Validated measures implemented consistently?	Mesaurement device clearly reported?	Protocol clearly described?	Sensor attachment reported?	Length of real-world assessment clearly described?	> 3 days of collected data controlled for?	Appropriate ethics and consent?	Were the statistical tests appropriate?	Probability values reported for the main outcomes?	Confounding variables statistically adjusted?	Reporting of results adequate	Average score
Corra, et al., 2021	R 1	Y	Y	Y	Y	Y	Y	Y	N	N	N	Y	Y	Y	Y	Y	12
	R 2	Y	Y	Y	Y	Y	Y	Y	N	Y	N	Y	Y	N	N	Y	11
Galperin, et al., 2019	R 1	Y	Y	Y	Y	Y	Y	Y	Y	Y	N	Y	Y	N	Y	N	12
	R 2	Y	Y	Y	Y	Y	Y	Y	Y	Y	Y	Y	N	N	N	N	11
Kirk, et al., 2023	R 1	Y	Y	Y	Y	Y	Y	Y	Y	Y	Y	Y	Y	Y	Y	Y	15
	R 2	Y	Y	Y	Y	Y	Y	Y	Y	Y	Y	Y	Y	Y	Y	Y	15
Mirleman n, et al., 2024	R 1	Y	Y	Y	Y	Y	Y	Y	Y	Y	Y	Y	Y	N	Y	Y	14
	R 2	Y	Y	Y	Y	Y	Y	Y	Y	Y	Y	Y	Y	N	Y	Y	14
Sarforpo ur, et al., 2022	R 1	N	Y	Y	Y	Y	Y	N	Y	N	N	Y	Y	Y	Y	Y	11
	R 2	Y	Y	Y	Y	Y	Y	Y	Y	Y	N	Y	Y	Y	N	Y	13
Shah, et al., 2020a	R 1	Y	Y	Y	Y	Y	Y	Y	N	Y	N	Y	Y	Y	N	Y	12
	R 2	Y	Y	Y	Y	Y	Y	Y	Y	Y	Y	Y	Y	Y	Y	Y	15
Terashi, et al., 2020	R 1	Y	Y	Y	Y	N	Y	Y	Y	Y	N	Y	Y	Y	Y	Y	12
	R 2	Y	Y	Y	Y	Y	Y	Y	Y	Y	N	Y	N	N.5	Y	Y	12
Terashi, et al., 2013	R 1	Y	N	Y	Y	N	Y	Y	Y	Y	N	N	N	N	N	Y	9
	R 2	Y	Y	Y	Y	N	Y	N	N	Y	N	Y	N	N	N	Y	8
Toosizad eh, et al., 2015	R 1	Y	Y	Y	N	Y	N	N	Y	Y	N	Y	Y	N	N	Y	9
	R 2	Y	Y	Y	Y	Y	N	N	Y	Y	N	Y	Y	N	N	Y	10

‘R’ denotes reviewer

Supplementary Table 6. Differences identified in the measurement methods of the supervised studies

**Instrument**	**n (%)**
Wearable device (lower back)	2 (33%)
Wearable device (lower back and feet)	1 (16%)
Wearable device (feet)	2 (33%)
Wearable device (shank, thigh and lower back)	1 (16%)
**Measurement task**	
Straight walk	3 (50%)
Curvilinear walking	1 (16%)
Straight and curvilinear walking	2 (33%)
**Single or dual task?**	
Single	5 (83%)
Single and dual task	1 (16%)
**Walking speed**	
Self-selected	3 (50%)
Self-selected and fast walking	2 (33%)
Did not specify	1 (16%)

Supplementary Table 7. EMBASE and MEDLINE Search strategy applied in the systematic review.

String no.	Query
#1. (Gait terms)	(((step* OR stride*) NEAR/2 (speed OR velocit* OR time* OR length* OR width* OR frequenc* OR rate* OR rhythm* OR variabilit* OR symmetr* OR asymmetr* OR count* OR number* OR distance* OR cadence*)):ti,ab) OR (((swing* OR stance* OR 'single support' OR 'double support') NEAR/2 (time* OR duration* OR variabilit* OR symmetr* OR asymmetr*)):ti,ab) OR (((spatiotemporal OR 'spatiotemporal') NEAR/2 (parameter* OR feature* OR characteristic*)):ti,ab) OR (((gait OR walk* OR ambulat*) NEAR/2 (speed OR velocit* OR time* OR cadence* OR pace* OR rhythm* OR volume* OR bout* OR duration* OR distance* OR intensit* OR variabilit* OR asymmetr* OR symmetr* OR parameter* OR feature* OR characteristic* OR assess* OR examin* OR analys* OR batter* OR measure* OR test*)) home OR domestic OR ((free OR daily) NEAR/2 living) OR ‘real -world’ OR ‘real world’ OR ‘community ambulat*’ OR (((day* OR daily OR ambulat* OR physical OR walk* OR monitor*) NEAR/2 activit*)) OR (((day* OR daily OR count* OR time OR number*) NEAR/2 (walk* OR step*)) OR ((sensor* OR record* OR monitor*) NEAR/2 (continu* OR activit* OR ‘long-term’ OR ‘long term’)) OR (Body NEAR/2 sensor*) OR Pedometer* OR *phone* OR (mobile NEAR/2 device*) :ti,ab)
#2 (Disease area terms)	'Parkinson disease'/exp OR 'parkinsonism' (parkinson* OR 'paralysis agitans')
#3 (Final)	#1 AND #2 AND (1999:py OR 2000:py OR 2001:py OR 2002:py OR 2003:py OR 2004:py OR 2005:py OR2006:py OR 2007:py OR 2008:py OR 2009:py OR 2010:py OR 2011:py OR 2012:py OR 2013:py OR 2014:py OR 2015:py OR 2016:py OR 2017:py OR 2018 :py OR 2019:py OR 2020:py OR 2021:py OR 2022:py OR 2023:py OR 2024:py)

Supplementary Table 8. CINHL Search strategy applied in the systematic review.

String no.	Query
#1. (Gait terms)	((step* OR stride*) N2 (speed OR velocit* OR time* OR length* OR width* OR frequenc* OR rate* OR rhythm* OR variabilit* OR symmetr* OR asymmetr* OR count* OR number* OR distance* OR cadence*) OR (swing* OR stance* OR 'single support' OR 'double support') N2 (time* OR duration* OR variabilit* OR symmetr* OR asymmetr*) OR (spatiotemporal OR 'spatiotemporal') N2 (parameter* OR feature* OR characteristic*) OR (gait OR walk* OR ambulat*) N2 (speed OR velocit* OR time* OR cadence* OR pace* OR rhythm* OR volume* OR bout* OR duration* OR distance* OR intensit* OR variabilit* OR asymmetr* OR symmetr* OR parameter* OR feature* OR characteristic* OR assess* OR examin* OR analys* OR batter* OR measure* OR test* AND (home OR domestic OR (free OR daily) N2 living OR 'real -world' OR 'real world' OR 'community ambulat*' OR (day* OR daily OR ambulat* OR physical OR walk* OR monitor*) N2 activit* OR (day* OR daily OR count* OR time OR number*) N2 (walk* OR step*) OR (sensor* OR record* OR monitor*) N2 (continu* OR activit* OR 'long-term' OR 'long term' OR Body N2 sensor* OR Pedometer* OR *phone* OR (mobile N2 device*))) Title
#2 (Disease area terms)	('Parkinson disease' OR 'parkinsonism' OR parkinson* OR 'paralysis agitans') Abstract

Supplementary Table 9. CINAHL Search strategy applied in the systematic review.

String no.	Query
#1. (Gait terms)	(("step*" OR "stride*") W/5 ("speed" OR "velocit*" OR "time*" OR "length*" OR "width*" OR "frequenc*" OR "rate*" OR "rhythm*" OR "variabilit*" OR "symmetr*" OR "asymmetr*" OR "count*" OR "number*" OR "distance*" OR "cadence*") OR ("swing*" OR "stance*" OR 'single support' OR 'double support') W/5 ("time*" OR "duration*" OR "variabilit*" OR "symmetr*" OR "asymmetr*") OR ("spatiotemporal" OR 'spatiotemporal') W/2 ("parameter*" OR "feature*" OR "characteristic*") OR ("gait" OR "walk*" OR "ambulat*") W/2 ("speed" OR "velocit*" OR "time*" OR "cadence*" OR "pace*" OR "rhythm*" OR "volume*" OR "bout*" OR "duration*" OR "distance*" OR "intensit*" OR "variabilit*" OR "asymmetr*" OR "symmetr*" OR "parameter*" OR "feature*" OR "characteristic*" OR "assess*" OR "examin*" OR "analys*" OR "batter*" OR "measure*" OR "test*") AND ("home" OR "domestic" OR ("free" OR "daily") W/2 "living" OR “real-world” OR “real world”) OR ("day*" OR "daily" OR "ambulat*" OR "physical" OR "walk*" OR "monitor*") W/2 "activit*" OR ("day*" OR "daily" OR "count*" OR "time" OR "number*") W/2 ("walk*" OR "step*") OR ("sensor*" OR "record*" OR "monitor*") W/2 ("continu*" OR "activit*" OR “long-term” OR “long term” OR "Body" W/2 "sensor*" OR "Pedometer*" OR "*phone*" OR ("mobile" AND "device*"))
#2 (Disease area terms)	AND (“Parkinson disease” OR “parkinsonism”))

Supplementary Table 10. Web of Science search strategy applied in the systematic review.

String no.	Query
#1. (Gait terms)	(((step* OR stride*) NEAR/2 (speed OR velocit* OR time* OR length* OR width* OR frequenc* OR rate* OR rhythm* OR variabilit* OR symmetr* OR asymmetr* OR count* OR number* OR distance* OR cadence*))) OR (((swing* OR stance* OR “single support” OR “double support”) NEAR/2 (time* OR duration* OR variabilit* OR symmetr* OR asymmetr*))) OR (((spatiotemporal OR 'spatio-temporal') NEAR/2 (parameter* OR feature* OR characteristic*))) OR (((gait OR walk* OR ambulat*) NEAR/2 (speed OR velocit* OR time* OR cadence* OR pace* OR rhythm* OR volume* OR bout* OR duration* OR distance* OR intensit* OR variabilit* OR asymmetr* OR symmetr* OR parameter* OR feature* OR characteristic* OR assess* OR examin* OR analys* OR batter* OR measure* OR test*))) AND ("home" OR "domestic" OR ("free" OR "daily") NEAR/2 "living" OR “real-world” OR “real world”) OR ("day*" OR "daily" OR "ambulat*" OR "physical" OR "walk*" OR "monitor*") NEAR/2 "activit*" OR ("day*" OR "daily" OR "count*" OR "time" OR "number*") NEAR/2 ("walk*" OR "step*") OR ("sensor*" OR "record*" OR "monitor*") NEAR/2 ("continu*" OR "activit*" OR “long-term” OR “long term” OR "Body") AND ("sensor*" OR "Pedometer*" OR "*phone*" OR ("mobile" AND "device*"))
#2 (Disease area terms)	AND (((“Parkinson disease” OR “parkinsonism”)))

Supplementary Table 11. IEEE explore search strategy applied in the systematic review.

String no.	Query
#1. (Gait terms)	(walk OR walking OR step OR speed OR gait OR spatiotemporal OR stride OR swing OR stance OR "single support" OR "double support" OR ambulation OR ambulatory)
#2 (Disease area terms)	AND (“Parkinson disease” OR “parkinsonism”)

Supplementary Table 12. Table of data extraction methods for the full-text review

	**Associated questions**
**Publication details**	
Authors and affiliations	Who conducted the research?
Type	In what type of literature was the study published (Journal, grey literature, conference abstract)
Year	When was the study published?
Country/region	In which geographic region(s) did the study take place?
**General details**	
Study design	What was the study's design?
Study aims	What were the study's aims?
Population	What population was studied? Were there any specific inclusion/exclusion criteria such as disease severity, subtype, or age?
Included DMOS	Which DMOs were measured?
**Technical details**	
Measurement device	How many devices were included? Which anatomical position were the device(s) worn?<br>How were the devices attached?<br>Who was the device manufactured by?
Real-world measurement protocol	How many days were participants assessed for?<br>Which aggregation method was applied?
**Assessment differences**	
Study setting	Were DMO quantitatively assessed during both unsupervised, continuous real-world assessment and supervised, scripted assessment in a clinic/laboratory environment, in the same study?
Differences in DMOs	What differences in DMOs occurred (or did not occur) between assessment condition?<br>Did these differences reach statistical

	significance?
**PD compared to controls**	
Study design	Were patients and controls matched or are the groups comparable with respect to appropriate criteria (height, age, sex)? Was gait analysis controlled for walking speed? Did the study focus on a specific subgroup or population?
Differences in DMOs	What differences in DMOs occurred (or did not occur) between people with PD and healthy controls? Did these differences reach statistical significance?
**Associations with motor severity**	
Analytical methods	How did the authors measure the relationship between motor severity and DMOs? What association measure was used?
Clinically relevant measures	
Relationship strength	What was the strength of the reported relationship between the measure and the DMO? Was the association statistically significant?
**Systematic Review**	
Risk of bias (quality assessment)	What is risk of bias and quality of the included articles?
Assessment of heterogeneity	Were the studies heterogenous in number of outcomes such as: population, intervention, technology used, assessment method, length of assessment and others.
Summary effect size	Within each study what is the effect size relative to each RQ?
Publication Bias	What proportion of the studies reported statistically significant or clinically favourable results?

Supplementary Table 13. Quality appraisal assessment

Category	Aspect of quality
External validity	• Was the study population clearly specified and defined? (age/gender/condition)<br>• Were inclusion and exclusion criteria for participants defined?<br>• Was the research question or objective in this paper clearly stated?
Internal validity	• Were the main outcomes clearly described in the methods/introduction.<br>• Could it be replicated?<br>• Validated measures (criterion/convergent/discriminant validity) and implemented consistently across all study participants?<br>• Devices for a data acquisition clearly reported?<br>• Protocol clearly described<br>• Sensor attachment reported?<br>• Length of real-world assessment clearly described?<br>• > 3 days of collected data controlled for?<br>• Appropriate ethics and consent?
Analysis	• Were the statistical tests used to assess the main outcomes appropriate (i.e. parametric vs. non-parametric)?<br>• Probability values reported (e.g. 0.026 rather than <0.05) for the main outcomes.<br>• Were key potential confounding variables measured and adjusted statistically for their impact on the outcome(s)?<br>• Was reporting of results adequate (i.e. no selective reporting)

Supplementary Table 14. Completed PRIMSA checklist of this systematic review.

Section and Topic	Item #	Checklist item	Location where item is reported
**TITLE**			
Title	1	Identify the report as a systematic review.	Title page
**ABSTRACT**			
Abstract	2	See the PRISMA 2020 for Abstracts checklist.	Abstract page
**INTRODUCTION**			
Rationale	3	Describe the rationale for the review in the context of existing knowledge.	Introduction
Objectives	4	Provide an explicit statement of the objective(s) or question(s) the review addresses.	Introduction
**METHODS**			
Eligibility criteria	5	Specify the inclusion and exclusion criteria for the review and how studies were grouped for the syntheses.	Methods
Information sources	6	Specify all databases, registers, websites, organisations, reference lists and other sources searched or consulted to identify studies. Specify the date when each source was last searched or consulted.	Methods
Search strategy	7	Present the full search strategies for all databases, registers and websites, including any filters and limits used.	Methods
Selection process	8	Specify the methods used to decide whether a study met the inclusion criteria of the review, including how many reviewers screened each record and each report retrieved, whether they worked independently, and if applicable, details of automation tools used in the process.	Methods
Data collection process	9	Specify the methods used to collect data from reports, including how many reviewers collected data from each report, whether they worked independently, any processes for obtaining or confirming data from study investigators, and if applicable, details of automation tools used in the process.	Methods
Data items	10a	List and define all outcomes for which data were sought. Specify whether all results that were compatible with each outcome domain in each study were sought (e.g. for all measures, time points, analyses), and if not, the methods used to decide which results to collect.	Methods
	10b	List and define all other variables for which data were sought (e.g. participant and intervention characteristics, funding sources). Describe any assumptions made about any missing or unclear information.	Methods
Study risk of bias assessment	11	Specify the methods used to assess risk of bias in the included studies, including details of the tool(s) used, how many reviewers assessed each study and whether they worked independently, and if applicable, details of automation tools used in the process.	Methods
Effect measures	12	Specify for each outcome the effect measure(s) (e.g. risk ratio, mean difference) used in the synthesis or presentation of results.	Methods
Synthesis methods	13a	Describe the processes used to decide which studies were eligible for each synthesis (e.g. tabulating the study intervention characteristics and comparing against the planned groups for each synthesis (item #5)).	Methods

Section and Topic	Item #	Checklist item	Location where item is reported
	13b	Describe any methods required to prepare the data for presentation or synthesis, such as handling of missing summary statistics, or data conversions.	Methods
	13c	Describe any methods used to tabulate or visually display results of individual studies and syntheses.	Methods
	13d	Describe any methods used to synthesize results and provide a rationale for the choice(s). If meta-analysis was performed, describe the model(s), method(s) to identify the presence and extent of statistical heterogeneity, and software package(s) used.	Methods
	13e	Describe any methods used to explore possible causes of heterogeneity among study results (e.g. subgroup analysis, meta-regression).	Methods
	13f	Describe any sensitivity analyses conducted to assess robustness of the synthesized results.	Methods
Reporting bias assessment	14	Describe any methods used to assess risk of bias due to missing results in a synthesis (arising from reporting biases).	Methods
Certainty assessment	15	Describe any methods used to assess certainty (or confidence) in the body of evidence for an outcome.	Methods
**RESULTS**			
Study selection	16a	Describe the results of the search and selection process, from the number of records identified in the search to the number of studies included in the review, ideally using a flow diagram.	Results
	16b	Cite studies that might appear to meet the inclusion criteria, but which were excluded, and explain why they were excluded.	Results
Study characteristics	17	Cite each included study and present its characteristics.	Results
Risk of bias in studies	18	Present assessments of risk of bias for each included study.	Results and Supplementary material
Results of individual studies	19	For all outcomes, present, for each study: (a) summary statistics for each group (where appropriate) and (b) an effect estimate and its precision (e.g. confidence/credible interval), ideally using structured tables or plots.	Results
Results of syntheses	20a	For each synthesis, briefly summarise the characteristics and risk of bias among contributing studies.	Results and Supplementary material
	20b	Present results of all statistical syntheses conducted. If meta-analysis was done, present for each the summary estimate and its precision (e.g. confidence/credible interval) and measures of statistical heterogeneity. If comparing groups, describe the direction of the effect.	Results
	20c	Present results of all investigations of possible causes of heterogeneity among study results.	Results
	20d	Present results of all sensitivity analyses conducted to assess the robustness of the synthesized results.	Not conducted; reasons provided

Section and Topic	Item #	Checklist item	Location where item is reported
			in Discussion (e.g., insufficient number of studies, heterogeneous data)
Reporting biases	21	Present assessments of risk of bias due to missing results (arising from reporting biases) for each synthesis assessed.	Not assessed; qualitative synthesis only
Certainty of evidence	22	Present assessments of certainty (or confidence) in the body of evidence for each outcome assessed.	Not assessed; limitations noted in Discussion
**DISCUSSION**			
Discussion	23a	Provide a general interpretation of the results in the context of other evidence.	Discussion
	23b	Discuss any limitations of the evidence included in the review.	Discussion
	23c	Discuss any limitations of the review processes used.	Discussion
	23d	Discuss implications of the results for practice, policy, and future research.	Discussion
**OTHER INFORMATION**			
Registration and protocol	24a	Provide registration information for the review, including register name and registration number, or state that the review was not registered.	Abstract page
	24b	Indicate where the review protocol can be accessed, or state that a protocol was not prepared.	Methods
	24c	Describe and explain any amendments to information provided at registration or in the protocol.	Amendments to protocol section
Support	25	Describe sources of financial or non-financial support for the review, and the role of the funders or sponsors in the review.	Acknowledgements and competing interests
Competing interests	26	Declare any competing interests of review authors.	Competing interests
Availability of data, code and other materials	27	Report which of the following are publicly available and where they can be found: template data collection forms; data extracted from included studies; data used for all analyses; analytic code; any other materials used in the review.	Complete

References

1. Abujrida, H., Agu, E. & Pahlavan, K. DeePaGait: Motor Assessment of Parkinson's Disease Using a Multi-Layer 1D Convolutional Neural Network on Smartphone Gait Data. in *2022 IEEE International Conference on Big Data (Big Data)* 5153–5162 (2022). doi:10.1109/BigData55660.2022.10021029.
2. Aich, S. *et al.* Design of a Machine Learning-Assisted Wearable Accelerometer-Based Automated System for Studying the Effect of Dopaminergic Medicine on Gait Characteristics of Parkinson's Patients. *J Healthc Eng* **2020**, 1823268 (2020).
3. Alazeb, A. *et al.* Effective Gait Abnormality Detection in Parkinson's Patients for Multi-Sensors Surveillance System. *IEEE Access* **12**, 48686–48698 (2024).
4. Atrsaei, A. *et al.* Gait speed in clinical and daily living assessments in Parkinson's disease patients: performance versus capacity. *NPJ PARKINSONS DISEASE* **7**, (2021).
5. Atrsaei, A. *et al.* Effect of Fear of Falling on Mobility Measured During Lab and Daily Activity Assessments in Parkinson's Disease. *Front Aging Neurosci* **13**, 722830 (2021).
6. Balakrishnan, A., Medikonda, J., Namboothiri, P. K. & Natarajan, M. Mahalanobis Metric-based Oversampling Technique for Parkinson's Disease Severity Assessment using Spatiotemporal Gait Parameters. *Biomedical Signal Processing and Control* **86**, 105057 (2023).
7. Barry, G. *et al.* Non-motor symptoms are associated with change in physical activity over 18 months in incident Parkinson's disease (PD): 18th International Congress of Parkinson's Disease and Movement Disorders (Movement Disorders 2014). in (2014).
8. Beigi, O. M., Nóbrega, L. R., Houghten, S., de Oliveira Andrade, A. & Pereira, A. A. Classification of Parkinson's Disease Patients and Effectiveness of Medication for Freezing of Gait. in *2022 IEEE Conference on Computational Intelligence in Bioinformatics and Computational Biology (CIBCB)* 1–8 (2022). doi:10.1109/CIBCB55180.2022.9863050.
9. Bernad-Elazari, H. *et al.* Objective characterization of daily living transitions in patients with Parkinson's disease using a single body-fixed sensor. *J Neurol* **263**, 1544–1551 (2016).

10. Bianchini, E. *et al.* Step-Counting Accuracy of a Commercial Smartwatch in Mild-to-Moderate PD Patients and Effect of Spatiotemporal Gait Parameters, Laterality of Symptoms, Pharmacological State, and Clinical Variables. *Sensors (Basel)* **23**, 214 (2022).

11. Bianchini, E. *et al.* Four Days Are Enough to Provide a Reliable Daily Step Count in Mild to Moderate Parkinson's Disease through a Commercial Smartwatch. *Sensors (Basel)* **23**, 8971 (2023).

12. Bobić, V., Đurić-Jovičić, M., Dragaševic-Mišković, N., Kostić, V. S. & Kvaščev, G. Comparison of Two Deep Learning Models for the Recognition of Parkinson's Disease Gait Patterns. in *2023 10th International Conference on Electrical, Electronic and Computing Engineering (IcETRAN)* 1–5 (2023). doi:10.1109/IcETRAN59631.2023.10192156.

13. Borzì, L., Sigcha, L. & Olmo, G. Context Recognition Algorithms for Energy-Efficient Freezing-of-Gait Detection in Parkinson's Disease. *Sensors (Basel)* **23**, 4426 (2023).

14. Brand, Y. E. *et al.* Gait Detection from a Wrist-Worn Sensor Using Machine Learning Methods: A Daily Living Study in Older Adults and People with Parkinson's Disease. *Sensors* **22**, 7094 (2022).

15. Brink-Kjær, A., Wickramaratne, S. D., Parekh, A. & During, E. H. Detection and Characterization of Walking Bouts Using a Single Wrist-Worn Accelerometer in Free-living Conditions. *medRxiv* 2023.08.01.23293509 (2023) doi:10.1101/2023.08.01.23293509.

16. Burq, M. *et al.* Virtual exam for Parkinson's disease enables frequent and reliable remote measurements of motor function. *npj Digit. Med.* **5**, 65 (2022).

17. Busse, M. E., Pearson, O. R., Van Deursen, R. & Wiles, C. M. Quantified measurement of activity provides insight into motor function and recovery in neurological disease. *J Neurol Neurosurg Psychiatry* **75**, 884–888 (2004).

18. Caballol, N., Bayés, À., Prats, A., Martín-Baranera, M. & Quispe, P. Feasibility of a wearable inertial sensor to assess motor complications and treatment in Parkinson's disease. *PLoS One* **18**, e0279910 (2023).

19. Cai, G. *et al.* Specific Distribution of Digital Gait Biomarkers in Parkinson's Disease Using Body-Worn Sensors and Machine Learning. *The Journals of Gerontology: Series A* **78**, 1348–1354 (2023).

20. Cesarelli, G. *et al.* Ataxia and Parkinson's disease patients classification using tree-based machine learning algorithms fed by spatiotemporal features: a pilot study. in *2022 IEEE International Symposium on Medical Measurements and Applications (MeMeA)* 1–6 (2022). doi:10.1109/MeMeA54994.2022.9856460.

21. Chen, O. Y. *et al.* Building a Machine-Learning Framework to Remotely Assess Parkinson's Disease Using Smartphones. *IEEE Trans Biomed Eng* **67**, 3491–3500 (2020).

22. Cheng, W.-Y. *et al.* Smartphone-based continuous mobility monitoring of Parkinsons disease patients reveals impacts of ambulatory bout length on gait features. in *2017 IEEE Life Sciences Conference (LSC)* 166–169 (2017). doi:10.1109/LSC.2017.8268169.

23. Cheng, W.-Y. *et al.* Large-Scale Continuous Mobility Monitoring of Parkinson's Disease Patients Using Smartphones. in *Wireless Mobile Communication and Healthcare* (eds. Perego, P., Rahmani, A. M. & TaheriNejad, N.) 12–19 (Springer International Publishing, Cham, 2018). doi:10.1007/978-3-319-98551-0_2.

24. Cherukuri, N., Jean, J. & McCalla, S. Leveraging Logistic Regression in Development of a Parkinson's Disease (PD) Kinematics-based Diagnostic Framework. in *2022 12th International Conference on Information Technology in Medicine and Education (ITME)* 693–698 (2022). doi:10.1109/ITME56794.2022.00146.

25. Cohen, M. *et al.* Multidisciplinary Intensive Rehabilitation Program for People with Parkinson's Disease: Gaps between the Clinic and Real-World Mobility. *Int J Environ Res Public Health* **20**, 3806 (2023).

26. Moulaee Conradsson, D., Leavy, B., Hagströmer, M. & Franzén, E. Predictors of Sustained Physical Activity During the COVID-19 Pandemic in People With Parkinson Disease in Sweden. *J Neurol Phys Ther* **48**, 75–82 (2024).

27. Cook, D. J., Schmitter-Edgecombe, M. & Dawadi, P. Analyzing Activity Behavior and Movement in a Naturalistic Environment Using Smart Home Techniques. *IEEE J Biomed Health Inform* **19**, 1882–1892 (2015).

28. D'Arco, L., Wang, H. & Zheng, H. A Rapid Detection of Parkinson's Disease using Smart Insoles: A Statistical and Machine Learning Approach. in *2022 IEEE International Conference on Bioinformatics and Biomedicine (BIBM)* 2985–2992 (2022). doi:10.1109/BIBM55620.2022.9995237.

29. da Rosa Tavares, J. E. *et al.* uTUG: An unsupervised Timed Up and Go test for Parkinson's disease. *Biomedical Signal Processing and Control* **81**, 104394 (2023).

30. de Faria, J. *et al.* Multicomponent and mat Pilates training increased gait speed in individuals with Parkinson's disease when walking and carrying a load: A single-blinded randomized controlled trial. *Physiother Res Int* **28**, e2031 (2023).

31. Del Din, S. *et al.* Continuous Real-World Gait Monitoring in Idiopathic REM Sleep Behavior Disorder. *J Parkinsons Dis* **10**, 283–299 (2020).

32. Dinesh, R. K. B., Surya, P. & Priya, B. Detection Approach Using Machine Learning for Parkinson's Disease. in *2022 1st International Conference on Computational Science and Technology (ICCST)* 1–5 (2022). doi:10.1109/ICCST55948.2022.10040313.

33. Divyashree, P. & Dwivedi, P. AI Computing as Ubiquitous Healthcare Solution: Predict Parkinson's for Large Masses in Society. *IEEE Transactions on Computational Social Systems* **10**, 1752–1757 (2023).

34. Domingues, V. L., Pompeu, J. E., de Freitas, T. B., Polese, J. & Torriani-Pasin, C. Physical activity level is associated with gait performance and five times sit-to-stand in Parkinson's disease individuals. *Acta Neurol Belg* **122**, 191–196 (2022).

35. Elavaar Kuzhali S *et al.* Analysis of Motor Skills for Parkinson's Disease Diagnosis. in *2023 OITS International Conference on Information Technology (OCIT)* 23–28 (2023). doi:10.1109/OCIT59427.2023.10430597.

36. El-Gohary, M. *et al.* Continuous Monitoring of Turning in Patients with Movement Disability. *Sensors* **14**, 356–369 (2014).

37. Escamilla-Luna, O., Wister, M. A. & Hernandez-Torruco, J. Classification Algorithms for Analyzing Parkinson's Disease Patient. in *2022 International Conference on Software, Telecommunications and Computer Networks (SoftCOM)* 1–6 (2022). doi:10.23919/SoftCOM55329.2022.9911391.

38. Galperin, I. *et al.* Sensor-Based and Patient-Based Assessment of Daily-Living Physical Activity in People with Parkinson's Disease: Do Motor Subtypes Play a Role? *Sensors (Basel)* **20**, 7015 (2020).

39. Ganesh D, Anupam Kumar Gautam, & Pawan Bhambu. Multimodal Data Fusion and Machine Learning for Comprehensive Management of Parkinson's Disease in Healthcare. in *2023 IEEE International Conference on ICT in Business Industry & Government (ICTBIG)* 1–7 (2023). doi:10.1109/ICTBIG59752.2023.10456100.

40. Ginis, P. *et al.* Validation of Commercial Activity Trackers in Everyday Life of People with Parkinson's Disease. *Sensors (Basel)* **23**, 4156 (2023).

41. Goh, C. H., Koh, C. H., Chong, Y. Z. & Lim, W. Y. Gait classification of parkinson's disease with supervised machine learning approach. in *7th IEEE-EMBS Conference on Biomedical Engineering and Sciences (IECBES 2022)* 112–116 (IEEE, Institute of Electrical and Electronics Engineers, 2022). doi:10.1109/IECBES54088.2022.10079640.

42. Gong, Y., Wang, Y., Wang, Z., Li, X. & Gu, Y. Longitudinal Analysis of Step Counts in Parkinson's Disease Patients: Insights from a Web-Based Application. 2023.11.22.23298898 Preprint at https://doi.org/10.1101/2023.11.22.23298898 (2023).

43. Goni, M., Eickhoff, S., Sahandi Far, M., Patil, K. R. & Dukart, J. Smartphone-Based Digital Biomarkers for Parkinson's Disease in a Remotely-Administered Setting. *28361 - 28384* **10**, 28361 (2836).

44. Hao, T. *et al.* An Automated Digital Biomarker of Mobility. in *2023 IEEE International Conference on Digital Health (ICDH)* 100–108 (2023). doi:10.1109/ICDH60066.2023.00022.

45. Haruyama, T., Zin, T. T., Sakai, K. & Mochizuki, H. A Study on Diagnosis of Parkinson's Disease by Walking Video. in *2021 IEEE 10th Global Conference on Consumer Electronics (GCCE)* 758–759 (2021). doi:10.1109/GCCE53005.2021.9621777.

46. Hausdorff, J. M. GAIT DYNAMICS, FRACTALS AND FALLS: FINDING MEANING IN THE STRIDE-TO-STRIDE FLUCTUATIONS OF HUMAN WALKING. *Hum Mov Sci* **26**, 555–589 (2007).

47. Herman, T., Weiss, A., Brozgol, M., Giladi, N. & Hausdorff, J. M. Gait and balance in Parkinson's disease subtypes: objective measures and classification considerations. *J Neurol* **261**, 2401–2410 (2014).

48. Ho, M.-Y. *et al.* Pathological Gait Analysis With an Open-Source Cloud-Enabled Platform Empowered by Semi-Supervised Learning-PathoOpenGait. *IEEE J Biomed Health Inform* **28**, 1066–1077 (2024).

49. Hobert, M. A. *et al.* Progressive Gait Deficits in Parkinson's Disease: A Wearable-Based Biannual 5-Year Prospective Study. *Front Aging Neurosci* **11**, 22 (2019).

50. Holleran, C. L. *et al.* Day-to-day Variability of Walking Performance Measures in Individuals Post-stroke and Individuals with Parkinson Disease. *J Neurol Phys Ther* **44**, 241–247 (2020).

51. Horak, F, McNames, J, Nutt, J, & Curtze, C. Classifying Parkinsonian Gait and Turning in Daily Life with Wearable Technology. in *MDS Abstracts* (Movement Disorders, Hong Kong, 2018).

52. Ingelse, L. *et al.* Personalised Gait Recognition for People with Neurological Conditions. *Sensors (Basel)* **22**, 3980 (2022).

53. Jiang, W. *et al.* Short Step Length Estimation for Parkinson's Disease Patients by Using Fusion Data From Camera-IMU in Smart Glasses. *IEEE Trans Biomed Eng* **71**, 2265–2275 (2024).

54. Kataoka, H. & Sugie, K. Health-Related Quality of Life May Predict Disease Staging in 10 Years in Parkinson Disease With Hoehn-Yahr Stage III. *Neurol Clin Pract* **11**, e261–e266 (2021).

55. Kegelmeyer, D. A. *et al.* Use of a Robotic Walking Device for Home and Community Mobility in Parkinson Disease: A Randomized Controlled Trial. *J Neurol Phys Ther* **48**, 102–111 (2024).

56. Kirk, C. *et al.* Mobilise-D insights to estimate real-world walking speed in multiple conditions with a wearable device. *Sci Rep* **14**, 1754 (2024).

57. Laurent, L. *et al.* Tinetti balance performance is associated with mortality in older adults with late-onset Parkinson's disease: a longitudinal study. *BMC Geriatr* **23**, 54 (2023).

58. Leavy, B *et al.* Clinical effectiveness of a highly challenging balance training (HiBalance program) for Parkinson's disease: A multi-center clinical effectiveness trial. in *MDS Abstracts* (Movement Disorders, Hong Kong, 2018).
59. Leavy, B., Hagströmer, M., Conradsson, D. M. & Franzén, E. Physical Activity and Perceived Health in People With Parkinson Disease During the First Wave of Covid-19 Pandemic: A Cross-sectional Study From Sweden. *J Neurol Phys Ther* **45**, 266–272 (2021).
60. Li, Y. *et al.* An abnormal gait monitoring system for patients with Parkinson's disease based on wearable devices. in *2022 15th International Congress on Image and Signal Processing, BioMedical Engineering and Informatics (CISP-BMEI)* 1–6 (2022). doi:10.1109/CISP-BMEI56279.2022.9980005.
61. Li, X., Lv, C., Liu, X. & Qin, X. Effects of Health Qigong Exercise on Lower Limb Motor Function in Parkinson's Disease. *Front Med (Lausanne)* **8**, 809134 (2022).
62. Li, W., Zhu, W., Dorsey, E. R. & Luo, J. Remote Medication Status Prediction for Individuals with Parkinson's Disease using Time-series Data from Smartphones. Preprint at https://doi.org/10.48550/arXiv.2207.13700 (2023).
63. Lin, C.-H. *et al.* Early Detection of Parkinson's Disease by Neural Network Models. *IEEE Access* **10**, 19033–19044 (2022).
64. Liu, Y. *et al.* Monitoring gait at home with radio waves in Parkinson's disease: A marker of severity, progression, and medication response. *Sci Transl Med* **14**, eadc9669 (2022).
65. López-Delgado, I. E. *et al.* mm-Wave wireless radar network for early detection of Parkinson's Disease by gait analysis. in *2023 IEEE Radar Conference (RadarConf23)* 1–6 (2023). doi:10.1109/RadarConf2351548.2023.10149746.
66. Lord, S. *et al.* Ambulatory activity in incident Parkinson's: more than meets the eye? *J Neurol* **260**, 2964–2972 (2013).
67. Mactier, K., Lord, S., Godfrey, A., Burn, D. & Rochester, L. The relationship between real world ambulatory activity and falls in incident Parkinson's disease: Influence of classification scheme. *Parkinsonism & Related Disorders* **21**, 236–242 (2015).

68. Malutan, R., Miclea, A. V., Barburiceanu, S. & Oltean, D. T. Data Analytics of Gait Monitoring from Parkinson's Disease Patients. in *2022 E-Health and Bioengineering Conference (EHB)* 1–4 (2022). doi:10.1109/EHB55594.2022.9991434.

69. Mancini, M. *et al.* The impact of freezing of gait on balance perception and mobility in community-living with Parkinson's disease. *Annu Int Conf IEEE Eng Med Biol Soc* **2018**, 3040–3043 (2018).

70. Mancini, M., Hasegawa, N., Peterson, D. S., Horak, F. B. & Nutt, J. G. Digital measures of freezing of gait across the spectrum of normal, non-freezers, possible freezers and definite freezers. *J Neurol* **270**, 4309–4317 (2023).

71. Mc Ardle, R. *et al.* Factors Influencing Habitual Physical Activity in Parkinson's Disease: Considering the Psychosocial State and Wellbeing of People with Parkinson's and Their Carers. *Sensors (Basel)* **22**, 871 (2022).

72. Mercuri, M. *et al.* A Tool for Home Monitoring in Parkinson's Disease. in *2022 5th International Conference on Advanced Communication Technologies and Networking (CommNet)* 1–5 (2022). doi:10.1109/CommNet56067.2022.9993923.

73. Mitoma, H., Yoneyama, M. & Orimo, S. 24-hour recording of parkinsonian gait using a portable gait rhythmogram. *Intern Med* **49**, 2401–2408 (2010).

74. Moon, S. H., Soangra, R., Frames, C. W. & Lockhart, T. E. Three days monitoring of activities of daily living among young healthy adults and parkinson's disease patients. *Biomedical Sciences Instrumentation* **57**, 177–183 (2021).

75. Moradi, H., Roth, N., Seifer, A. & Eskofier, B. M. Detection of distorted gait and wearing-off phenomenon in Parkinson's disease patients during Levodopa therapy. in *2022 IEEE-EMBS International Conference on Biomedical and Health Informatics (BHI)* 01–04 (2022). doi:10.1109/BHI56158.2022.9926873.

76. Moradi, H. *et al.* Monitoring medication optimization in patients with Parkinson's disease. *Annu Int Conf IEEE Eng Med Biol Soc* **2023**, 1–4 (2023).

77. Morgan, C. *et al.* A multimodal dataset of real world mobility activities in Parkinson's disease. *Sci Data* **10**, 918 (2023).

78. Morris, R. *et al.* A model of free-living gait: A factor analysis in Parkinson's disease. *Gait & Posture* **52**, 68–71 (2017).

79. Morris, R, Martini, D, Mancini, M, & Horak, F. The interplay between cholinergic activity, attention, and turning in Parkinson's disease. in *MDS Abstracts* (Movement Disorders, Nice, 2019).

80. Naimi, S., Bouachir, W. & Bilodeau, G.-A. HCT: Hybrid Convnet-Transformer for Parkinson's disease detection and severity prediction from gait. Preprint at https://doi.org/10.48550/arXiv.2310.17078 (2023).

81. Negi, P. C., Negi, S. & Sharma, N. Gait Analysis-Based Identification of Neurodegenerative Diseases Using Machine Learning Techniques. in *2022 International Conference on Advances in Computing, Communication and Materials (ICACCM)* 1–6 (2022). doi:10.1109/ICACCM56405.2022.10009413.

82. Nouriani, A., Jonason, A., Jean, J., McGovern, R. & Rajamani, R. System-Identification-Based Activity Recognition Algorithms With Inertial Sensors. *IEEE Journal of Biomedical and Health Informatics* **27**, 3119–3128 (2023).

83. Olmos, J. & Martinez, F. A Riemannian Deep Learning Representation to Describe Gait Parkinsonian Locomotor Patterns. *Annu Int Conf IEEE Eng Med Biol Soc* **2022**, 3538–3541 (2022).

84. Otlet, V. & Ronsse, R. Predicting the effects of oscillator-based assistance on stride-to-stride variability of Parkinsonian walkers. in *2022 International Conference on Robotics and Automation (ICRA)* 8083–8089 (2022). doi:10.1109/ICRA46639.2022.9811822.

85. Oyama, G. *et al.* Analytical and clinical validity of wearable, multi-sensor technology for assessment of motor function in patients with Parkinson's disease in Japan. *Sci Rep* **13**, 3600 (2023).

86. Pallavi, P. *et al.* Design of SmartWalk for Estimating Implication of Pathway With Turn and Task Condition on Postural and Gait Indices: Relevance to Fear of Fall. *IEEE Trans Neural Syst Rehabil Eng* **31**, 406–415 (2023).

87. Patil, K. S., George, S. M., Naik, K. G., Chethana, P. & Kamath, N. V. Freeze of Gait and Fall Detection in Parkinson's Patients. in *2022 4th International Conference on Circuits, Control, Communication and Computing (I4C)* 245–249 (2022). doi:10.1109/I4C57141.2022.10057615.

88. Pelicioni, P. H. S. *et al.* Combined Reactive and Volitional Step Training Improves Balance Recovery and Stepping Reaction Time in People With Parkinson's Disease: A Randomised Controlled Trial. *Neurorehabil Neural Repair* **37**, 694–704 (2023).

89. Pham, T. D. Visual Concurrent Analysis of Gait Patterns Among Healthy Young, Old Adults, and Patients With Parkinson's Disease. in *2022 IEEE International Conference on Bioinformatics and Biomedicine (BIBM)* 2674–2681 (2022). doi:10.1109/BIBM55620.2022.9995627.

90. Pisal, A., Agarwal, B. M. & Mullerpatan, R. Evaluation of Daily Walking Activity in Patients with Parkinson Disease. *CRP* **30**, (2018).

91. Porta, M. *et al.* Association between Objectively Measured Physical Activity and Gait Patterns in People with Parkinson's Disease: Results from a 3-Month Monitoring. *Parkinsons Dis* **2018**, 7806574 (2018).

92. Prusynski, R. A., Kelly, V. E., Fogelberg, D. J. & Pradhan, S. The association between sleep deficits and sedentary behavior in people with mild Parkinson disease. *Disabil Rehabil* **44**, 5585–5591 (2022).

93. Pullagura, L. *et al.* ML based Parkinson's Disease Identification using Gait Parameters. in *2022 International Conference on Automation, Computing and Renewable Systems (ICACRS)* 561–566 (2022). doi:10.1109/ICACRS55517.2022.10029281.

94. Rabuffetti, M. *et al.* Association of 7-Day Profiles of Motor Activity in Marital Dyads with One Component Affected by Parkinson's Disease. *Sensors* **23**, 1087 (2023).

95. Rastegari, E., Marmelat, V., Najjar, L., Bastola, D. & Ali, H. H. Using gait parameters to recognize various stages of Parkinson's disease. in *2017 IEEE International Conference on Bioinformatics and Biomedicine (BIBM)* 1647–1651 (2017). doi:10.1109/BIBM.2017.8217906.

96. Rastegari, E., Ali, H. & Marmelat, V. Detection of Parkinson's Disease Using Wrist Accelerometer Data and Passive Monitoring. *Sensors (Basel)* **22**, 9122 (2022).

97. Raykov, Y. P. *et al.* Probabilistic Modelling of Gait for Robust Passive Monitoring in Daily Life. *IEEE J Biomed Health Inform* **25**, 2293–2304 (2021).

98. Rochester, L., Chastin, S. F. M., Lord, S., Baker, K. & Burn, D. J. Understanding the impact of deep brain stimulation on ambulatory activity in advanced Parkinson's disease. *J Neurol* **259**, 1081–1086 (2012).

99. Roth, N. *et al.* Do We Walk Differently at Home? A Context-Aware Gait Analysis System in Continuous Real-World Environments. in *2021 43rd Annual International Conference of the IEEE Engineering in Medicine & Biology Society (EMBC)* 1932–1935 (2021). doi:10.1109/EMBC46164.2021.9630378.

100. Shah, V *et al.* Association between Gait during Daily Life and Clinical Measures: Effects of Bout Length. in *MDS Abstracts* (Movement Disorders, Nice, 2019).

101. Shah, V. V. *et al.* Inertial Sensor Algorithms to Characterize Turning in Neurological Patients With Turn Hesitations. *IEEE Transactions on Biomedical Engineering* **68**, 2615–2625 (2021).

102. Shah, V *et al.* Comparison of gait measures in a clinic and a community setting in people with and without Parkinson's disease. in *MDS Abstracts* (Virtual, 2020).

103. Shih, H.-J. S. *et al.* Utilization of wearable activity monitors to support physical activity interventions in neurodegenerative diseases: a feasibility study. 2022.05.31.22275824 Preprint at https://doi.org/10.1101/2022.05.31.22275824 (2022).

104. Shih, H.-J. S. *et al.* Wearable activity monitors to support physical activity interventions in neurodegenerative disease: a feasibility study. *Neurodegener Dis Manag* **13**, 177–189 (2023).

105. Shimada, H. *et al.* Identification of Disability Risk in Addition to Slow Walking Speed in Older Adults. *Gerontology* **68**, 625–634 (2021).

106. Shokouhi, N., Khodakarami, H., Fernando, C., Osborn, S. & Horne, M. Accuracy of Step Count Estimations in Parkinson's Disease Can Be Predicted Using Ambulatory Monitoring. *Front Aging Neurosci* **14**, 904895 (2022).

107. Silva de Lima, A. L. *et al.* Impact of motor fluctuations on real-life gait in Parkinson's patients. *Gait Posture* **62**, 388–394 (2018).

108. Silva, R. do N. *et al.* Dual-task intervention based on trail making test: Effects on Parkinson's disease. *J Bodyw Mov Ther* **27**, 628–633 (2021).

109. Singh, N. & Tripathi, P. Gait Assessment using Optimized Machine Learning and Feature Selection Algorithm for identifies Parkinson's Disease. in *2023 IEEE International Students' Conference on Electrical, Electronics and Computer Science (SCEECS)* 1–6 (2023). doi:10.1109/SCEECS57921.2023.10062964.

110. Sorici, A., Băjenaru, L., Mocanu, I. & Florea, A. M. An Intelligent Ecosystem for Improving Brain Disease Monitoring of Patients Using Wearable Devices and Artificial Intelligence. in *2023 24th International Conference on Control Systems and Computer Science (CSCS)* 452–459 (2023). doi:10.1109/CSCS59211.2023.00077.

111. Stuckenschneider, T. *et al.* Disease-inclusive exercise classes improve physical fitness and reduce depressive symptoms in individuals with and without Parkinson's disease—A feasibility study. *Brain Behav* **11**, e2352 (2021).

112. Sturchio, A. *et al.* Kinematic but not clinical measures predict falls in Parkinson-related orthostatic hypotension. *J Neurol* **268**, 1006–1015 (2021).

113. Sütçü, G., Ayvat, E. & Kilinç, M. Effects of fatigue and kinesiophobia on functional capacity, physical activity and quality of life in Parkinson's disease. *Int J Rehabil Res* **44**, 65–68 (2021).

114. Terashi, H, Mitoma, H, Taguchi, T, & Aizawa, H. Relationship between daily physical activity measured by a triaxial accelerometer and motor symptoms in patients with Parkinson's disease. in *MDS Abstracts* (Movement Disorders, Nice, 2019).

115. Terashi, H., Ueta, Y., Taguchi, T., Mitoma, H. & Aizawa, H. Clinical Features of Parkinson's Disease in Patients with Early-Onset Freezing of Gait. *Parkinsons Dis* **2022**, 4732020 (2022).

116. Terashi, H. *et al.* Deficits in Scaling of Gait Force and Cycle in Parkinsonian Gait Identified by Long-Term Monitoring of Acceleration with the Portable Gait Rhythmogram. *ISRN Neurol* **2012**, 306816 (2012).

117. Tian, H. *et al.* Cross-Spatiotemporal Graph Convolution Networks for Skeleton-Based Parkinsonian Gait MDS-UPDRS Score Estimation. *IEEE Trans Neural Syst Rehabil Eng* **32**, 412–421 (2024).

118. Tigrini, A. *et al.* Gait Event Timeseries Assessment through Spectral Biomarkers and Machine Learning. in *2023 IEEE 36th International Symposium on Computer-Based Medical Systems (CBMS)* 257–262 (2023). doi:10.1109/CBMS58004.2023.00227.

119. Tsakanikas, V. D. *et al.* Gait and balance patterns related to Free-Walking and TUG tests in Parkinson's Disease based on plantar pressure data. *Annu Int Conf IEEE Eng Med Biol Soc* **2021**, 236–239 (2021).

120. Tucak, C., Chih, H., Mastaglia, F. & Rodrigues, J. The 'PD Warrior' exercise programme improves motor outcomes and quality of life in patients with early Parkinson disease: results of a pilot study. *Intern Med J* **54**, 823–832 (2024).

121. Ullrich, M. *et al.* Detection of Unsupervised Standardized Gait Tests From Real-World Inertial Sensor Data in Parkinson's Disease. *IEEE Trans Neural Syst Rehabil Eng* **29**, 2103–2111 (2021).

122. Utsumi, H. *et al.* How far do the complaints of patients with Parkinson's disease reflect motor fluctuation? Quantitative analysis using a portable gait rhythmogram. *ISRN Neurol* **2012**, 372030 (2012).

123. Utsumi, H. *et al.* Quantitative Assessment of Gait Bradykinesia in Parkinson's Disease Using a Portable Gait Rhythmogram. *Acta Medica Okayama* **66**, 31–40 (2012).

124. A, V. B. S. & M, P. R. Diagnosis of Neurodegenerative Diseases by Gait Analysis using Triblock CNN and Deep RQA Techniques. in *2022 International Conference on Automation, Computing and Renewable Systems (ICACRS)* 762–770 (2022). doi:10.1109/ICACRS55517.2022.10029144.

125. Vila, M. H., Pérez, R., Mollinedo, I. & Cancela, J. M. Analysis of Gait for Disease Stage in Patients with Parkinson's Disease. *Int J Environ Res Public Health* **18**, 720 (2021).

126. von der Recke, F., Warmerdam, E., Hansen, C., Romijnders, R. & Maetzler, W. Reduced Range of Gait Speed: A Parkinson's Disease-Specific Symptom? *J Parkinsons Dis* **13**, 197–202 (2023).

127. von Rosen, P., Hagströmer, M., Franzén, E. & Leavy, B. Physical activity profiles in Parkinson's disease. *BMC Neurol* **21**, 71 (2021).

128. Waddell, K. J. *et al.* Deploying Digital Health Technologies for Remote Physical Activity Monitoring of Rural Populations With Chronic Neurologic Disease. *Arch Rehabil Res Clin Transl* **5**, 100250 (2023).

129. Wells, M. D. *et al.* Walk with Me Hybrid Virtual/In-Person Walking for Older Adults with Neurodegenerative Disease. *J Vis Exp* (2023) doi:10.3791/62869.

130. Williamson, J. R., Telfer, B., Mullany, R. & Friedl, K. E. Detecting Parkinson's Disease from Wrist-Worn Accelerometry in the U.K. Biobank. *Sensors (Basel)* **21**, 2047 (2021).

131. Winkler, P., DeMarch, E., Campbell, H. & Smith, M. Use of real-time multimodal sensory feedback home program improved backward stride and retention for people with Parkinson Disease: A pilot study. *Clin Park Relat Disord* **6**, 100132 (2022).

132. Wu, X. *et al.* A Wearable Multi-sensor System for Classification of Multiple System Atrophy and Parkinson's Disease. in *2022 10th International Conference on Bioinformatics and Computational Biology (ICBCB)* 129–134 (2022). doi:10.1109/ICBCB55259.2022.9802460.

133. PD-ResNet for Classification of Parkinson's Disease From Gait. *IEEE J Transl Eng Health Med* **10**, 2200111 (2022).

134. Yogev-Seligmann, G. *et al.* The development of a home-based technology to improve gait in people with Parkinson's disease: a feasibility study. *BioMedical Engineering OnLine* **22**, 2 (2023).

135. Yokote, A. *et al.* Leg Muscle Strength Correlates with Gait Performance in Advanced Parkinson Disease. *Intern Med* **61**, 633–638 (2022).

136. Yoneyama, M., Kurihara, Y., Watanabe, K. & Mitoma, H. Accelerometry-based gait analysis and its application to Parkinson's disease assessment- part 2: a new measure for quantifying walking behavior. *IEEE Trans Neural Syst Rehabil Eng* **21**, 999–1005 (2013).

137. Yoneyama, M. *et al.* Ambulatory Gait Behavior in Patients With Dementia: A Comparison With Parkinson's Disease. *IEEE Trans Neural Syst Rehabil Eng* **24**, 817–826 (2016).

138. Yue, P., Li, Z., Zhou, M., Wang, X. & Yang, P. Wearable-Sensor-Based Weakly Supervised Parkinson's Disease Assessment with Data Augmentation. *Sensors* **24**, 1196 (2024).

139. Zajac, J. A. *et al.* Does clinically measured walking capacity contribute to real-world walking performance in Parkinson's disease? *Parkinsonism Relat Disord* **105**, 123–127 (2022).

140. Zeng, X. Towards in-Home Quantitative Gait Assessment Using Millimeter-wave Radar. in *2023 IEEE MTT-S International Microwave Biomedical Conference (IMBioC)* 88–90 (2023). doi:10.1109/IMBioC56839.2023.10305133.

141. Zeng, Q. *et al.* Video-Based Quantification of Gait Impairments in Parkinson's Disease Using Skeleton-Silhouette Fusion Convolution Network. *IEEE Transactions on Neural Systems and Rehabilitation Engineering* **31**, 2912–2922 (2023).

142. Zhao, H. *et al.* Accurate identification of Parkinson's disease by distinctive features and ensemble decision trees. *Biomedical Signal Processing and Control* **69**, 102860 (2021).

143. Zhao, Y. *et al.* Intelligent IoT Anklets for Monitoring the Assessment of Parkinson's Diseases. *IEEE Sensors Journal* **23**, 31523–31536 (2023).

144. Zoetewei, D. *et al.* On-Demand Cueing for Freezing of Gait in Parkinson's Disease: A Randomized Controlled Trial. *Movement Disorders* **39**, 876–886 (2024).
